# A Highly Reversible Aqueous Sulfur‐Dual‐Halogen Battery Enabled by a Water‐in‐Bisalt Electrolyte

**DOI:** 10.1002/smll.202502228

**Published:** 2025-04-17

**Authors:** Ronghuan Liang, Yan Wang, Chuanlong Wei, Xiao Tang, Timing Fang, Zhezheng Ding, Qing Wang, Rui Wang, Jianjun Song, Bing Sun, Xiaomin Liu, Guoxiu Wang

**Affiliations:** ^1^ School of Chemistry and Chemical Engineering Qingdao University Qingdao Shandong 266071 P. R. China; ^2^ College of Physics Qingdao University Qingdao Shandong 266071 P. R. China; ^3^ Centre for Clean Energy Technology School of Mathematical and Physical Sciences Faculty of Science University of Technology Sydney Ultimo NSW 2007 Australia

**Keywords:** aqueous rechargeable batteries, chlorine‐based redox reaction, interhalogens coordinating chemistry, sulfur‐dual halogen chemistry, water‐in‐bisalt electrolyte

## Abstract

The chlorine‐based redox reaction applied in aqueous rechargeable batteries (ARBs) has attracted extensive attention owing to the high theoretical capacity and redox potential. However, it generally suffers from low reversibility and poor Coulombic efficiency due to the evolution of toxic Cl_2_ gas and the decomposition of aqueous electrolytes. Herein, an aqueous sulfur‐dual halogen chemistry is demonstrated by employing highly‐concentrated water‐in‐bisalt (WiBS) electrolyte, sulfur anode, and iodine composite electrodes. The freestanding iodine/carbon cloth cathode and Cl^−^‐containing WiBS electrolyte not only enable the continuous I^+^/I^0^ reaction by forming [ICl_x_]^1−x^ interhalogens but also achieve the oxidation of Cl^−^ in [ICl_x_]^1−x^ at higher redox potential and immobilize Cl^0^ species via I^+^─Cl^0^ chemical bonds. Therefore, the as‐assembled aqueous sulfur‐dual halogen batteries (ASHBs) based on the dual‐halogen conversion on the cathode and the S/S_x_^2−^ redox reaction on the anode deliver a high energy density of 304 Wh kg^−1^ with an average output voltage of 1.32 V. These key findings open an avenue for the development of low‐cost and high‐performance ARBs for energy storage applications.

## Graphical abstract

An aqueous sulfur‐dual halogen battery (ASHBs) has been developed by using Cl^−^‐containing water‐in‐bisalt (WiBS) electrolyte, iodine composite cathode, and sulfur/MXene composite anode. The as‐assembled ASHBs deliver a superior electrochemical performance based on dual‐halogen conversion reactions.

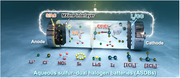

## 1 Introduction

Lithium‐ion batteries have been widely used in EVs and renewable energy storage owing to their high energy density (250–400 Wh kg^−1^).^[^ ^1^ ^]^ However, the high cost associated with the scarcity of lithium resources and safety concerns (i.e., combustion and explosion due to the usage of flammable organic electrolytes) has severely restrained their grid‐scale application.^[^ ^2^ ^]^ Meanwhile, aqueous rechargeable batteries (ARBs) have attracted considerable attention, offering distinctive advantages such as low cost, high safety, high ionic conductivity, and environmental friendliness.^[^ ^3^ ^]^ Nevertheless, the electrochemical stability window of aqueous electrolytes is limited due to the poor thermodynamics of water solvents, thereby restricting the employment of high‐voltage electrode materials.^[^ ^4^ ^]^ Furthermore, the current electrode materials used in ARBs (e.g., lithium manganese oxide, vanadium oxides, Prussian blue analogs, etc.) exhibit relatively low specific capacities.^[^ ^5^ ^]^ These inherent limitations in energy density and electrochemical stability could hinder future development of aqueous rechargeable batteries.

**Figure 1:**
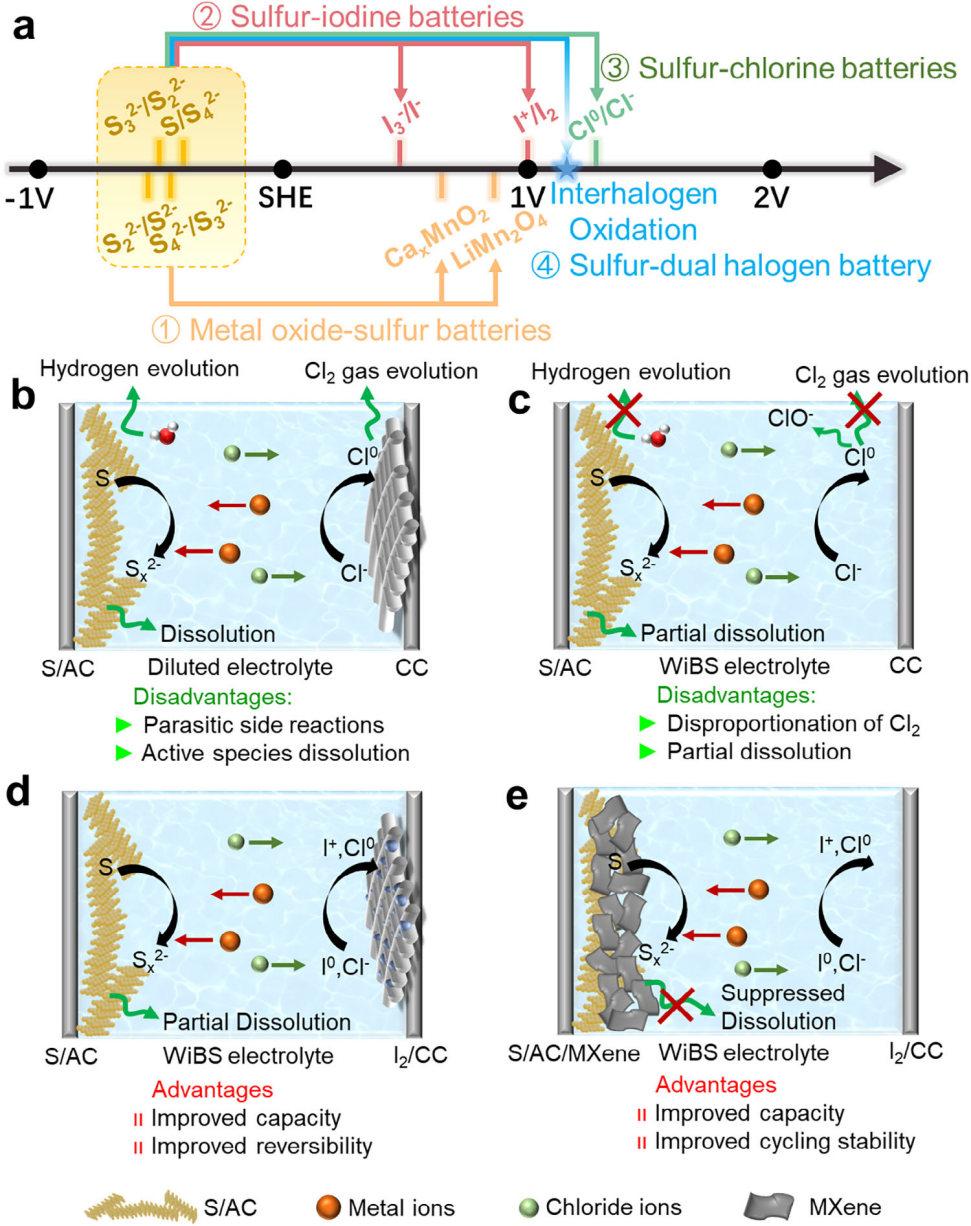
Schematic illustrations of the working mechanisms of the aqueous sulfur‐dual halogen battery. a) The schematic illustration of different potentials of redox couples in different battery configurations. b,c) The working mechanism and advantages/disadvantages of aqueous sulfur‐(dual) halogen batteries with different configurations.

To unlock the potential of high‐performance ARBs, novel battery chemistries need to be rationally designed by matching cathode/anode and aqueous electrolytes. For instance, elemental sulfur has been selected as an anode material owing to its low cost, environmental friendliness, simple manufacturing process, and high theoretical capacity (1672 mAh g^−1^).^[^ ^6^ ^]^ The employment of sulfur anode can fundamentally avoid the dendritic growth issue of metal anodes. Recently, aqueous sulfur‐metal oxide batteries have been developed based on sulfur composite anodes, which achieved an improved high output voltage of the battery system (**Figure** **1**a).^[^ ^6^, ^7^ ^]^ However, the cathodes (i.e., metal oxide) used in these studies usually exhibit low specific capacities (less than 300 mAh g^−1^), which leads to a low negative/positive ratio and limits the energy density of the entire aqueous batteries. Therefore, developing innovative cathodes with high specific capacities is critical to further enhancing the electrochemical performance of ARBs.

Compared with traditional intercalation‐type cathode materials, halogen‐based cathode materials exhibit significantly higher energy density due to their multi‐electron transfer mechanisms. Iodine‐based redox reactions (e.g., I^0^/I⁻, I^+^/I^0^, IO_3_^−^/I^−^, etc.) have recently garnered considerable attention.^[^ ^4^, ^8^ ^]^ Our previous development of aqueous S–I_2_ batteries has validated the sulfur‐halogen battery system. However, its capacity is still constrained by iodine redox reaction (211 mAh g^−1^ of I⁺/I^0^).^[^ ^8c^ ^]^ Additionally, bromine‐based redox couples (Br^0^/Br^−^, Br^+^/Br^0^) offer higher redox potential and theoretical capacity.^[^ ^9^ ^]^ Noticeably, the chlorine‐based redox reaction (CRR) possesses the highest theoretical capacity (755 mAh g^−1^), an acceptable redox potential (1.36 V vs standard hydrogen electrode (SHE)), and fast reaction kinetics with an activation energy of 35.5 kJ mol^−1^, making CRR as a promising cathode candidate for high‐energy‐density ARBs.^[^ ^10^ ^]^ Nevertheless, chlorine‐based redox reactions still suffer from some deficiencies. The high operation potential of CRR in aqueous electrolytes is usually accompanied by electrolyte decomposition, which accelerates performance deterioration. Additionally, the CRR exhibits inferior reversibility and low Coulombic efficiency (CE) due to the evolution of toxic Cl_2_ gas and the disproportionation of Cl_2_ in aqueous electrolyte, which may lead to the formation of hypochlorous acid and hypochlorite.^[^ ^11^ ^]^ Several strategies have been employed to improve the electrochemical performance of CRR. For instance, graphite material has been applied to host metal‐halide complex ions, but the irreversible structural disruption resulting from repeated intercalation may lead to rapid capacity decay during cycling.^[^ ^12^ ^]^ Another approach involves the reduction of the operation temperature (usually below the boiling point of Cl_2_, i.e., −34 °C), which allows the reversible electroplating/stripping of liquefied Cl_2_ on the host cathode.^[^ ^13^ ^]^ However, such harsh operation conditions may not align with the requirements for large‐scale practical application of high‐performance ARBs. Recently, I^+^ ions (generated from I^+^/I^0^ redox reaction) have been found to have the capability to bond with Cl^−^ ions and form solid‐state interhalogen, which is expected to benefit the chlorine‐based redox reactions in aqueous electrolytes.^[^ ^14^, ^15^ ^]^

In this work, we have developed a dual‐halogen conversion chemistry to achieve an aqueous sulfur‐dual halogen battery (ASHB), which consists of a sulfur/activated carbon/MXene (S/AC/MXene) anode, a water‐in‐bisalt (WiBS) electrolyte, and an iodine/carbon cloth (I_2_/CC) cathode. The S/AC/MXene anode has been applied, which not only provides high specific capacity and mitigates polysulfide dissolution, but also fundamentally avoids the dendritic growth issue of metal anodes. The highly‐concentrated WiBS aqueous electrolyte (15 m (mol kg^−1^_water_) LiCl‐7 m  LiNO_3_ aqueous solution) not only supplies chloride sources for the cathode redox reactions but also possesses reduced free water molecules, which can significantly suppress the electrolyte decomposition. At the cathode side, the I_2_/CC cathode first undergoes the I^+^/I^0^ redox reaction at lower voltage during the charging process, providing extra capacity and supplying I^+^ to stabilize Cl^−^ by forming interhalogen. Then, the generated I^+^ can effectively catalyze the reaction kinetics of Cl^0^/Cl^−^ redox reaction and immobilize the generated Cl^0^ with the formation of [ICl_2_]^0^ or [ICl_3_]^−^, therefore improving the reversibility of Cl^0^/Cl^−^ redox couple. The as‐assembled ASHBs can deliver a high specific capacity of 242 mAh g^−1^_total electrode_ (846 mAh g^−1^_sulfur_) with an average output voltage of 1.32 V and superior cycling stability. By synergizing multi‐electron transfer reaction design with electrolyte engineering, this work establishes a novel paradigm for next‐generation energy storage systems that combine high energy density, inherent safety, and cost‐effectiveness.

## 2 Results and Discussion

### 2.1 The Design Concept and Optimization Strategies of ASHBs

Figure 1 presents the schematic illustration of the optimization strategies for the ASHBs. Figure 1b shows the configuration of a general aqueous sulfur–chlorine battery that consists of an S/AC anode, a carbon cloth (CC) cathode, and a diluted electrolyte. During the charging process, the sulfur is reduced to polysulfides (S_x_^2−^). Meanwhile, the chloride ions from the electrolyte undergo an oxidation process to generate Cl^0^ on the cathode. The discharge process is the corresponding reverse reactions (S_x_^2−^ to S^0^ and Cl^0^ to Cl^−^). However, the disproportionation of Cl_2_ in aqueous electrolyte and evolution of toxic Cl_2_ gas at the cathode side, along with polysulfides dissolution into the electrolyte at the anode side, lead to poor cycling stability and low Coulombic efficiency of sulfur–chlorine batteries. In addition, due to the high operation voltage, such battery system suffers from severe electrolyte decomposition, leading to the rapid failure of the aqueous sulfur‐chlorine battery. By employing highly concentrated aqueous electrolytes (e.g., WiBS electrolyte), the parasitic side reactions (e.g., Cl_2_ gas evolution and water splitting) at the cathode side are expected to be suppressed or even eliminated, while the polysulfides dissolution at the anode side will be mitigated, which can effectively improve the reversibility of the entire battery system (Figure 1c).^[^ ^7^ ^]^ Additionally, solvated NO_3_^−^ ions will accumulate on the anode surface and decompose to form solid state interphase (SEI) layers in highly concentrated electrolytes.^[^ ^8c^ ^]^ This SEI layer prevents further NO_3_^−^ decomposition and protects the anode by mitigating polysulfide migration.

Although the employment of the WiBS electrolyte can improve the battery reversibility due to the reduced water solvent reactivity, the electrochemical stability still needs to be further optimized. A dual‐halogen conversion strategy is proposed to further immobilize Cl^0^ on the cathode via introducing iodine/carbon cloth as the cathode. The I_2_/CC cathode delivers a continuous multi‐electron redox reaction, where the iodine in the cathode undergoes an oxidation process from I^0^ to I^+^ in the initial charge process that tends to bond with Cl^−^ to form interhalogen ([ICl_x_]^1−x^). Then, Cl^−^ in the interhalogens can be further oxidized into Cl^0^. The introduction of iodine atoms not only improves the specific capacity of the cathode but also catalyzes the Cl^0^/Cl^−^ reaction and immobilizes Cl^0^ with the formation of [ICl_2_]^0^ or [ICl_3_]^−^, preventing the disproportionation reaction of Cl^0^ (Figure 1d). Furthermore, we also prepared sulfur/activated carbon/MXene (S/AC/MXene) composite to stabilize the sulfur anode. The 2D MXene (Ti_3_C_2_) with abundant surface terminations possesses good metallic conductivity, which is beneficial for achieving stable chemical interaction with polysulfides.^[^ ^16^ ^]^ The dissolution/diffusion of polysulfides can be effectively suppressed due to the protection of the MXene interlayer and the formation of SEI layers derived from nitrate anions reduction in the WiBS electrolyte (Figure 1e).^[^ ^17^ ^]^

### 2.2 Performance of WiBS Electrolyte in Aqueous Sulfur–Chlorine Batteries

**Figure 2:**
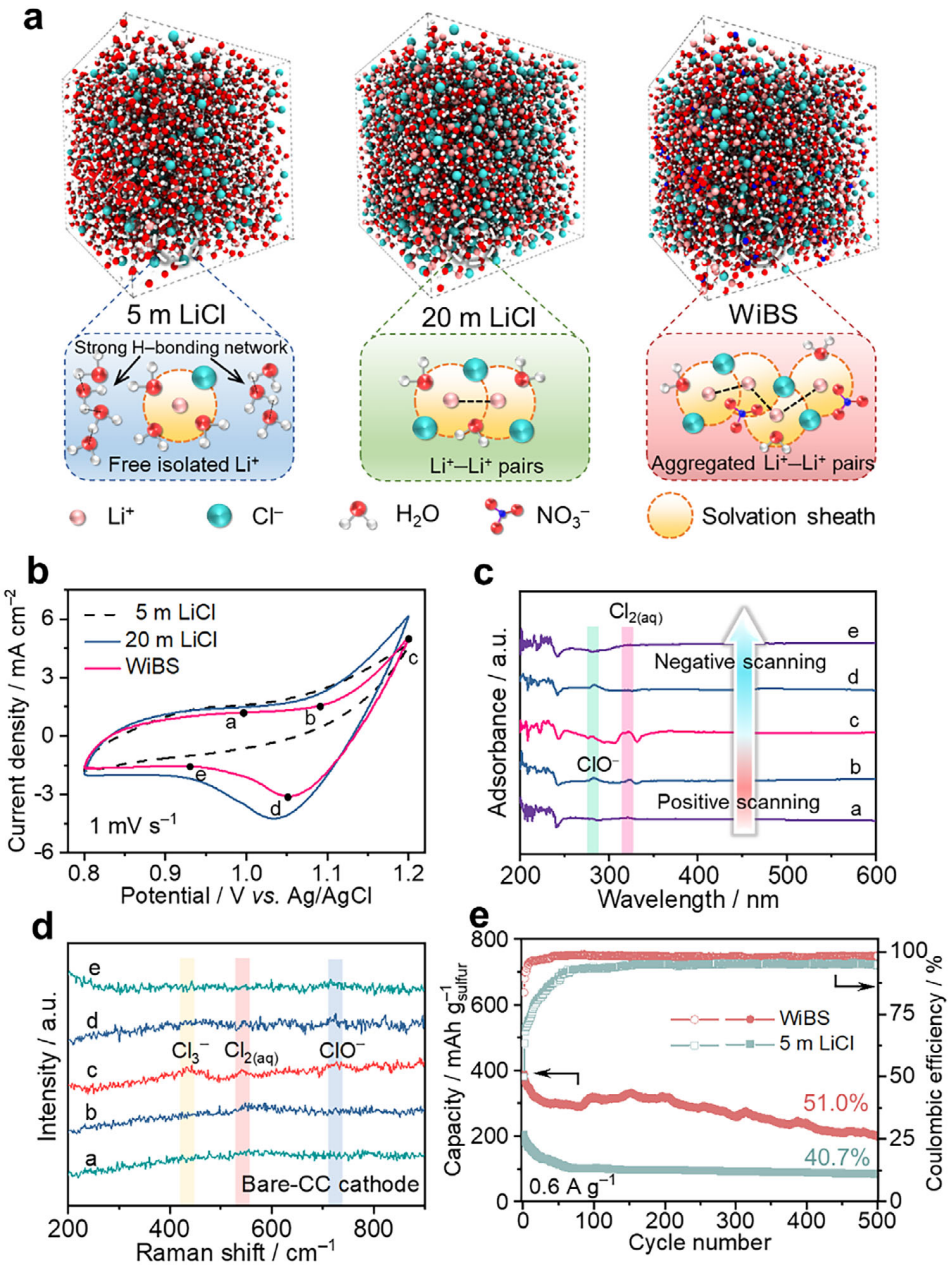
The working mechanism and electrochemically evaluation of Cl^0^/Cl^−^ conversion chemistry. a) The snapshots of MD simulation and the schematic diagram of solvation structures in 5 m  (left), 20 m  LiCl (middle), and WiBS (right) electrolytes. b) The CV curves of carbon cloth in different aqueous electrolytes at a scan rate of 1 mV s^−1^ via a three‐electrode configuration. c) The corresponding UV–vis spectra of the WiBS electrolytes immerging with carbon cloth at different states. d) The corresponding Raman spectra of carbon cloth at different states. e) The cycling performances and the corresponding Coulombic efficiencies of aqueous S/AC||CC cells with different electrolytes at 0.6 A g^−1^.

An aqueous electrolyte with 15 m  LiCl and 7 m  LiNO_3_ has been prepared, which not only stabilizes the electrophilic I^+^ via forming interhalogens but also suppresses the side reactions and restricts the diffusion of active species. **Figure** **2**a shows molecular dynamics (MD) simulation snapshot images and schematic diagrams of solvation structures of different aqueous electrolytes. In the low‐concentration electrolyte (5 m  LiCl aqueous solution), most water molecules interact with each other through a hydrogen bond network, inevitably triggering the decomposition of the water solvent at high voltage.^[^ ^18^ ^]^ In comparison, the bisalt strategy can break the constraints of individual solubility limits, thereby increasing the concentration of the aqueous electrolyte.^[^ ^19^ ^]^ Therefore, large amounts of Li^+^ tend to share the primary water sheaths with each other, and on average less than 2 water molecules are coordinated with each Li^+^.^[^ ^8c^ ^]^ This breaks the hydrogen bond network between water molecules and decreases the free water content, thus suppressing the side reaction associated with water splitting.^[^ ^20^ ^]^ This can also be confirmed by the lowest chemical shift of the WiBS electrolyte in ^1^H nuclear magnetic resonance spectroscopy (^1^H NMR) spectroscopy (Figure S1, Supporting Information).

The reversibility of Cl^0^/Cl^−^ redox reaction was first investigated in a three‐electrode configuration with a carbon cloth as a working electrode, a platinum wire as a counter electrode, and an Ag/AgCl electrode as the reference electrode. Figure 2b shows the cyclic voltammetry (CV) curves of pristine carbon cloth electrodes in various aqueous electrolytes. No obvious redox peak can be observed in the 5 m  LiCl electrolyte. Meanwhile, the transparent 5 m  LiCl electrolyte was transferred to a yellowish‐green solution after CV tests (Figure S2a, Supporting Information), which indicates an irreversible chloride conversion chemistry in the low‐concentration electrolyte. Intriguingly, with increasing the salt concentration more than 10 mol kg^−1^, the CV curves show obvious reduction peaks at ≈1.05 V versus Ag/AgCl, suggesting the improved reversibility of Cl^0^/Cl^−^ reaction (Figure 2b; Figure S3, Supporting Information). Noticeably, no significant color change was observed in the highly concentrated electrolyte. This further proves that the dissolution and diffusion of the generated Cl_2_ can be effectively suppressed in the highly concentrated electrolytes (right panel of Figure S2, Supporting Information).^[^ ^11b^ ^]^ When employing the WiBS electrolyte, CV curves exhibit an oxidation peak starting at ≈1.0 V versus Ag/AgCl during the initial anodic scan, suggesting the transformation of Cl^−^ into oxidized active species. This can be confirmed by the ex situ UV–vis spectroscopy (hydrated chlorine (Cl_2_) at 322 nm, pink region in Figure 2c).^[^ ^21^ ^]^ Noticeably, ClO^−^ at 284 nm can be detected (peaks in blue and pink regions in Figure 2c), which is a byproduct of the disproportionation reaction:^[^ ^22^ ^]^

(1) $$Cl_{2}+H_{2}O\;↔\;\mathrm{HClO}\;+\;\mathrm{HCl}$$

Furthermore, these species (i.e., Cl_3_^−^, Cl_2_, and ClO^−^) were also monitored in the final charge products based on the ex situ Raman spectroscopy results. As shown in Figure 2d, when the scanning potential reaches 1.2 V versus Ag/AgCl, two peaks at 425 and 541 cm^−1^ appear, corresponding to the generation of Cl_3_^−^ and hydrated chlorine, respectively.^[^ ^10^, ^23^ ^]^ Additionally, the signal of ClO^−^ also appears at 723 cm^−1^.^[^ ^11a^ ^]^ Noticeably, the formation of ClO^−^ may inevitably lead to the loss of active species for the cathodic redox reactions.

The Cl^0^/Cl^−^ conversion chemistry was further electrochemically evaluated via a S/AC||CC cell, which consists of a sulfur/activated carbon (S/AC) composite anode and a freestanding carbon cloth cathode. The morphology and material characterizations of S/AC anode materials are shown in Figure S4 (Supporting Information). Figure 2e and Figure S5 (Supporting Information) present the cycling performance and the corresponding Coulombic efficiencies of S/AC|5 m  LiCl|CC and S/AC|WiBS|CC cells. The full cells with the 5  LiCl electrolyte only deliver a discharge capacity of 204 mAh g^−1^ (based on the mass of sulfur) with low initial Coulombic efficiency (≈50%) and poor cycling performance (only 40.7% capacity retention after 500 cycles); Meanwhile, no obvious discharge plateau can be observed in the voltage profiles (Figure S5a, Supporting Information), suggesting that the full cell cannot be stably cycled in the low‐concentration aqueous electrolytes. In contrast, S/AC|WiBS|CC cells exhibit a higher initial capacity (389 mAh g^−1^ based on the mass of sulfur) and an average Coulombic efficiency of 97.9%. Since the high salt concentration and the formation of inorganic‐rich SEI on sulfur anode can suppress the migration of active species, enhanced cycling performance with ≈51% capacity retention can be achieved after 500 cycles in the WiBS electrolyte.^[^ ^8c^ ^]^ Noticeably, both active species from the cathode side and the anode side exhibit high solubility in an aqueous environment, the shuttle effect occurring at both sides eventually leads to rapid capacity decay during cycling.

### 2.3 The Conversion Mechanism of Cl^0^/Cl^−^ Redox on I_2_/CC Cathode in WiBS Electrolyte

To further increase the energy density on the cathode side, a dual‐halogen conversion strategy has been proposed, in which iodine atoms were introduced as coordinating agents to bond with Cl^−^ and Cl^0^.^[^ ^24^ ^]^ The freestanding iodine/carbon cloth (I_2_/CC) composite cathode was prepared via a solution‐adsorption method (see morphology and structure information in Figure S6, Supporting Information). Figure S7 (Supporting Information) shows the CV curves of carbon cloth‐based electrodes with/without iodine in the WiBS electrolytes. Compared with a pristine carbon cloth electrode, the I_2_/CC composite cathode shows an extra pair of redox peaks at 0.63/0.87 V versus Ag/AgCl, which can be ascribed to the reversible I^+^/I^0^ conversion reaction.^[^ ^25^ ^]^ Meanwhile, the I_2_/CC composite cathode presents stronger Cl^0^/Cl^−^ redox peaks at ≈1.0 V, showing significantly increased current density (4.42 mA cm^−2^) compared to that of carbon cloth electrodes. These results indicate that the generated I^+^ ions can bond with Cl^0^/Cl^−^ atoms to form interhalogens, which not only retain Cl^0^ in the solid state and suppress the formation of toxic Cl_2_,^[^ ^24^ ^]^ but also facilitate the reaction kinetics of CRR by stimulating the oxidation of interhalogens ([ICl_x_]^1−x^) instead of Cl^−^ in the bulk electrolyte.^[^ ^26^ ^]^

Noticeably, the stability of interhalogens is degraded by the I^+^ hydrolysis in low‐concentration electrolytes.^[^ ^14^ ^]^ By tuning the activity of H_2_O in the electrolyte solution, the hydrolysis can be effectively suspended, thereby improving the stability of interhalogens. The MD simulations were conducted to scrutinize the free water content in different aqueous electrolytes (Figure S8, Supporting Information). After increasing the salt concentration, the free water content significantly decreases from 63.05% in 5 m  LiCl electrolyte to 17.18% in 20 m  LiCl electrolyte. By employing bisalt strategy, the free water content further decreases to 14.56% in the WiBS electrolyte. Therefore, such a reduced free water amount not only mitigates the dissolution and diffusion of active substances (e.g., I^+^, Cl^0^, and S_x_^2−^ species) but also inhibits the electrolyte decomposition (e.g., water splitting), thus benefiting the stable electrochemical performances.

**Figure 3:**
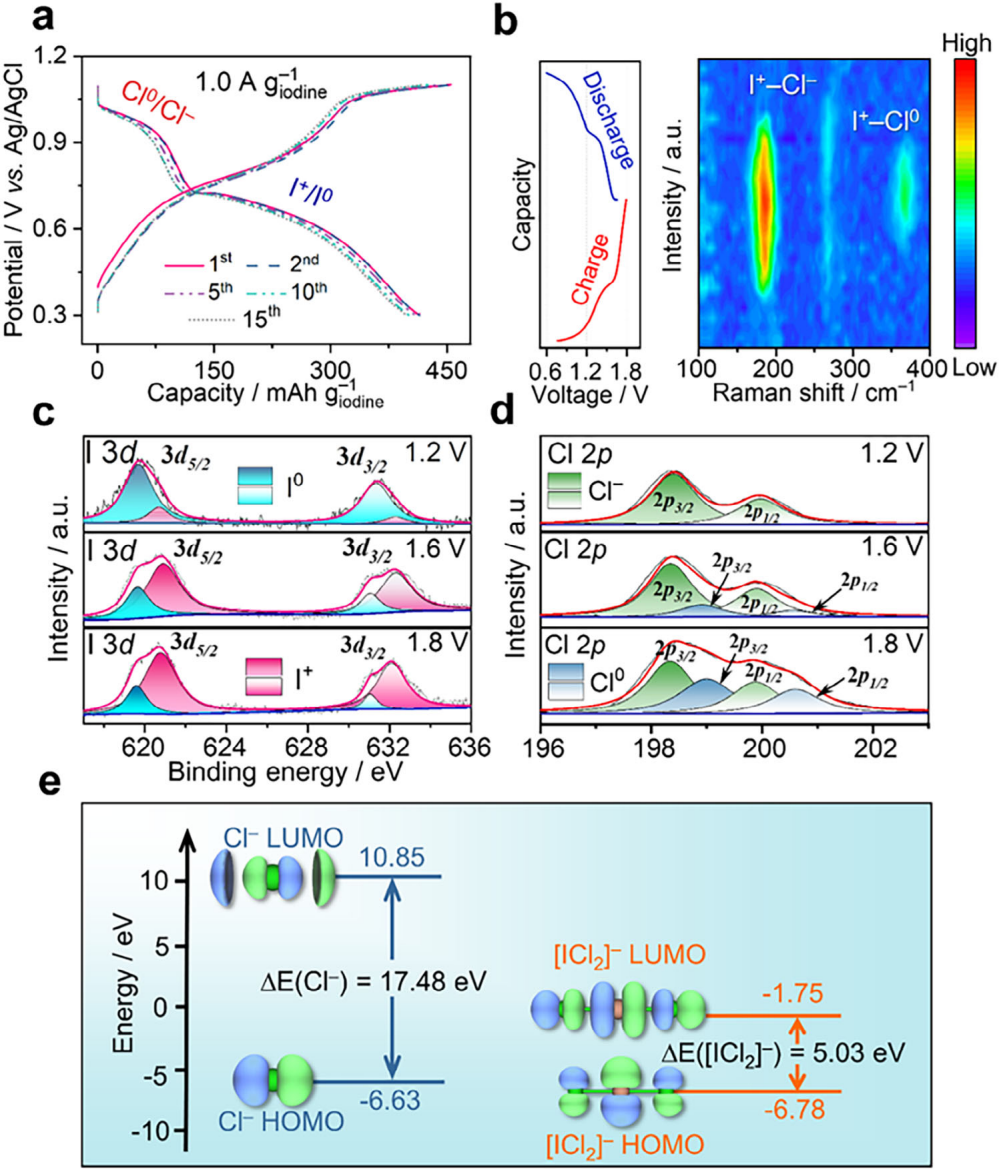
The reaction mechanism of the dual‐halogens conversion chemistry in I_2_/CC cathode. a) The voltage profiles of the I_2_/CC cathode in the WiBS electrolyte at a current density of 1.0 A g^−1^_iodine_ with the potential range of 0.3–1.1 V (vs Ag/AgCl). b) The voltage profile and contour plot of in situ Raman spectra of the I_2_/CC cathode in the range of 100–400 cm^−1^. The high‐resolution (c) I 3d and (d) Cl 2p XPS of I_2_/CC cathode disassembled from full cells at different voltage stages. e) The HOMOs, LUMOs, and energy gaps of Cl^−^ in bulk electrolyte and [ICl_2_]^−^ interhalogens.

The electrochemical behavior of the I_2_/CC composite cathode in the WiBS electrolyte was examined by galvanostatic charge/discharge via a three‐electrode configuration. **Figure** **3**a shows the voltage profiles of the I_2_/CC cathode at a current density of 1 A g^−1^, in which two discharge plateaus at ≈0.66 and ≈1.0 V (vs Ag/AgCl) can be observed, indicating the I^+^/I^0^ and Cl^0^/Cl^−^ conversion reactions, respectively. Based on the I^+^/I^0^ and Cl^0^/Cl^−^ conversion reactions, a high discharge capacity of 416 mAh g^−1^_iodine_ can be achieved. Noticeably, the I_2_/CC cathode delivers a specific capacity of ≈290 mAh g^−1^ within a voltage range of 0.3–0.9 V, exceeding the theoretical capacity of the I^+^/I^0^ redox couple (211 mAh g^−1^). This enhancement is primarily attributed to the partial contribution of the carbon cloth substrate, which exhibits capacitive behavior. Meanwhile, a high Coulombic efficiency (94.7% in the 15th cycle) and highly overlapped voltage profiles at different cycles suggest the superior reversibility of the redox reactions in the WiBS electrolyte. Additionally, by analyzing the CV curves at different scan rates, the capacity at the peak region is determined by both diffusion‐controlled and capacitive behaviors (See details in Figure S9, Supporting Information). We further evaluated the electrochemical performance of the I_2_/CC anode with a mass loading of 1 mg cm^−2^. Notably, a significantly lower areal capacity of 0.51 mAh cm^−2^ was obtained (Figure S10, Supporting Information), which is insufficient to match the high‐energy sulfur anode.

The conversion route of the composite cathode was investigated by ex situ X‐ray photoelectron spectroscopy (XPS), ex situ UV–vis, and in situ Raman measurements (Figure 3b–d; Figure S11, Supporting Information). Figure 3b and Figure S11 (Supporting Information) present the in situ Raman spectra during cycling. The peaks observed at ≈185 and 260 cm^−1^ corresponded to the [ICl_x_]^1−x^ interhalogens species.^[^ ^27^ ^]^ The intensity of these peaks gradually increases within the charge voltage range of 1.26–1.58 V, confirming the I^+^/I^0^ redox reaction. Noticeably, when charging the cell to 1.62 V, a peak at ≈358 cm^−1^ emerges, which should be attributed to the further oxidation of Cl^−^ in [ICl_x_]^1−x^ interhalogens.^[^ ^24^ ^]^ The peak intensities of interhalogen and Cl^0^ gradually weaken during the discharge process and disappear eventually, indicating good reversibility for the Cl^0^/Cl^−^ and I^+^/I^0^ redox reactions. Additionally, no newly formed peaks related to the formation of hydrated chlorine are detected (≈540 cm^−1^), which further confirms the efficient fixation of Cl^0^ by I^+^ species through interhalogens bonding chemistry (shown in Figure S11, Supporting Information).

The XPS measurements were carried out to further investigate the redox reactions in the I_2_/CC cathode. As presented in Figure 3c, the high‐resolution I 3*d* XPS spectra show two doublets at ≈619.7 and ≈631.4 eV, corresponding to the I 3*d_5/2_* and I 3*d_3/2_*, respectively.^[^ ^28^ ^]^ Both doublets can be deconvoluted into two sets of peaks with the I^0^ at 619.6/631.0 eV and the I^+^ at 620.8/632.1 eV.^[^ ^25^ ^]^ During the charge process, the intensities of I^+^ peaks gradually increase, while the I^0^ peaks show a significant reduction, which confirms the continuous conversion reaction from I^0^ to I^+^. Meanwhile, Figure 3d displays the Cl 2*p* XPS spectra during the charge process. Two doublets at 198.4 and 199.9 eV correspond to the Cl 2*p_3/2_* and Cl 2*p_1/2_*, respectively.^[^ ^29^ ^]^ Noticeably, the Cl^−^ signal is predominated till the cell was charged to 1.6 V, implying that Cl^−^ anions merely served as complexation agents for the formation of interhalogens at the low‐order charge plateau. By further charging the cell to 1.8 V, Cl^0^ peaks at 199.0/200.6 eV can be observed, which indicates the successful oxidation of the generated interhalogens at the higher‐order charge plateau.^[^ ^24^, ^30^ ^]^ This conversion mechanism can also be validated by UV–vis spectroscopy of the WiBS electrolyte by immersing the I_2_/CC cathode at different charge/discharge states (Figure S12, Supporting Information), in which an absorption peak appears at ≈227 nm in the fully charged state, which is related to the generation of Cl^0^ species, revealing the oxidation process from Cl^−^ to Cl^0^ in interhalogens.^[^ ^11b^ ^]^

Density functional theory (DFT) calculations were conducted to investigate the conformation of interhalogens. Figure S13 (Supporting Information) demonstrates the Gibbs free energy difference (△G) and optimized structure of possible interhalogens (e.g., ICl, [ICl_2_]^−^). Noticeably, the ICl can spontaneously transfer to [ICl_2_]^−^ in the bulk electrolyte, and the [ICl_2_]^−^ is expected to possess two optimized structures. The △G between [ICl_2_]^−^ (I–Cl–Cl) and ICl is 0.15 eV, indicating the thermodynamic instability of I–Cl–Cl conformation; Meanwhile, the [ICl_2_]^−^ (Cl–I–Cl) shows a negative △G (−0.58 eV), which suggests the spontaneous conversion from ICl to [ICl_2_]^−^ in the presence of Cl^−^. Therefore, [ICl_2_]^−^ interhalogen with Cl–I–Cl conformation is energetically more favorable as an intermediate product, which is similar to previously reported works.^[^ ^31^ ^]^ Figure S14 (Supporting Information) presents the difference in charge density of the [ICl_2_]^−^. It is found that the apparent charge transfer from the iodine atom to the chloride atom verifies the strong chemical interactions between iodine and chloride and further confirms the conformation of the generated interhalogens.

These results can be further confirmed by the frontier molecular orbital theory. Figure 3e presents the highest occupied molecular orbitals (HOMOs) and lowest unoccupied molecular orbitals (LUMOs) of Cl^−^ and [ICl_2_]^−^ (Cl–I–Cl). Noticeably, the Cl^−^ in WiBS electrolyte shows a wide energy gap of 17.48 eV, implying the strong stability toward its oxidation and relatively slow conversion kinetics of Cl^0^/Cl^−^. In contrast, a reduced energy gap of 5.03 eV can be observed in [ICl_2_]^−^ interhalogen, which suggests higher chemical reactivity. Therefore, the Cl^−^ in [ICl_2_]^−^ interhalogens is expected to preferentially oxidize to Cl^0^, which strongly implies the catalysis effect of I^+^. The calculation results are consistent with the CV testing results. The reaction that occurred at 1.60–1.80 V could be ascribed to the Equation (2) or (3):

(2) $${\left[\mathrm{IC}l_{2}\right]}^{-}\;-\;e^{-}\;↔\;{\left[\mathrm{IC}l_{2}\right]}^{0}$$

Or;

(3) $${\left[\mathrm{IC}l_{2}\right]}^{-}\;-\;e^{-}+\;Cl^{-}\;↔\;{\left[\mathrm{IC}l_{3}\right]}^{-}$$

This result can be further confirmed by the thermogravimetric analysis (TGA) of the I_2_/CC cathode disassembled from ASHBs at the fully charged state (see details in Figure S15, Supporting Information).

### 2.4 Electrochemical Performances of the As‐Developed ASHBs

To evaluate the electrochemical performance of ASHBs, Swagelok‐type full cells were assembled using S/AC anodes, I_2_/CC cathodes, and aqueous electrolytes. We also introduce an MXene interlayer to improve the cycling performance of S/AC anodes. The accordion‐like multi‐layered MXene with surface functional groups (─F and ─O) is shown in Figure S16 (Supporting Information). Figure S17a (Supporting Information) presents the CV curves of the S/AC anode with/without MXene interlayer in the WiBS electrolyte. The CV curve of the S/AC anode shows a pair of redox peaks at −0.67/−0.49 V versus Ag/AgCl, which can be ascribed to the reversible conversion of S/S_x_^2−^. After employing the MXene interlayer, the S/AC anode shows a reduced polarization, suggesting that the conversion reaction of S/S_x_^2−^ can be accelerated by MXene. Furthermore, the binding energies between Mxene, Li_2_S_4,_ and Li_2_S_6_ were investigated viaDFT calculations to confirm the chemical adsorption of MXene toward polysulfides. As shown in Figure S17b (Supporting Information), the binding energies of MXene with Li_2_S_4_ or Li_2_S_6_ are calculated to be −5.77 eV and −7.49 eV, respectively. This illustrates that MXene with abundant surface functional groups (i.e., ─OH) could provide a strong affinity to polysulfides, therefore mitigating the shuttling of active species.

**Figure 4:**
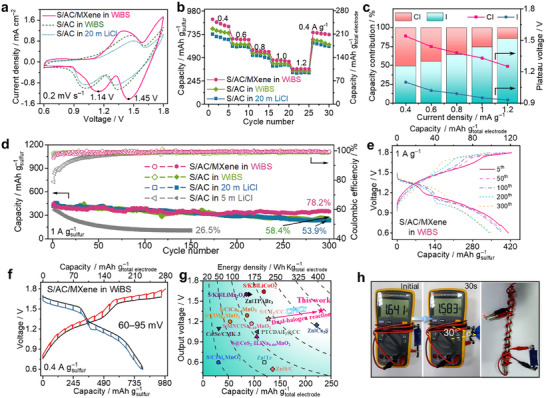
The electrochemical performances of the as‐assembled ASHBs. a) The CV curves of different ASHBs at the scan rate of 0.2 mV s^−1^. b) The rate performance of different ASHBs. c) The corresponding capacity contribution from Cl and I with discharge plateau voltage at different current densities in the S/AC/MXene|WiBS|I_2_/CC full cell. d) The cycling performances and corresponding Coulombic efficiencies of different ASHBs at 1 A g^−1^. The mass ratio of sulfur to iodine was set as 1:1. e) The voltage profiles of S/AC/MXene|WiBS|I_2_/CC full cell during 300 cycles at 1 A g^−1^. f) The GITT characterization of S/AC/MXene|WiBS|I_2_/CC full cell at 0.4 A g^−1^. The black curve is a quasi‐equilibrium potential of charge/discharge stages, which was constructed from the last data point of each open‐circuit voltage period. g) The comparison of output voltage, specific capacities, and energy densities (based on the total mass of the electrodes) of different energy storage devices based on the aqueous chalcogen and halogen conversion chemistry. h) The flexibility test of the ASHBs assembled by the self‐controlled insulating tape.

**Figures** **4**a and S18 (Supporting Information) present the CV curves of ASHBs at 0.2 mV s^−1^. By using the 20 m  LiCl electrolyte and the WiBS electrolyte, two pairs of redox peaks can be observed, which can be assigned to the I^+^/I^0^ and Cl^0^/Cl^−^ redox couples, respectively. The redox reactions of the cathode, anode, and overall electrochemical reactions are presented as follows:

Anode:

(4) $$S\;+2e^{-}\;↔\;\frac{1}{x}S_{x}^{2-}\;\left(x\;=1,2,\;\mathrm{etc}.\right)$$

Cathode:

Step 1:

(5) $$I_{2}+2\mathrm{yC}l^{-}\;-\;2e^{-}\;↔\;2{\left[\mathrm{IC}l_{y}\right]}^{1-y}\;\left(y\;=1,2,\;\mathrm{etc}.\right)$$

Step 2:

(6) $$2{\left[\mathrm{IC}l_{y}\right]}^{1-y}+2Cl^{-}\;-\;2e^{-}\;↔\;2{\left[\mathrm{IC}l_{1+y}\right]}^{1-y}$$

Overall:

(7) $$S\;+\frac{1}{2}I_{2}+\left(1+1y\right)Cl^{-}\;↔\;\frac{1}{x}S_{x}^{2-}+{\left[\mathrm{IC}l_{1+y}\right]}^{1-y}$$

Noticeably, the CV curves of ASHBs using 20 m  LiCl electrolyte and the WiBS electrolyte show high repeatability, indicating that the cathode and anode are highly reversible in highly concentrated electrolytes (Figure S18a,b, Supporting Information). By employing the MXene interlayer, ASHBs display redox peaks with enhanced current densities, which implies the adsorption ability of the MXene interlayer toward polysulfides (Figure S18c, Supporting Information). Furthermore, the decreased polarization indicates the enhanced redox kinetics of optimized ASHBs owing to the catalysis effect of MXene interlayer to the sulfur anodes, which is also consistent with the previously reported works.^[^ ^32^ ^]^

Figure 4b and Figures S19 and S20 (Supporting Information) present the rate performances, corresponding voltage profiles, and corresponding Coulombic efficiencies of ASHBs. The S/AC|20 m  LiCl|I_2_/CC cells can deliver 702, 606, 509, 407, and 329 mAh g^−1^ at current densities of 0.4, 0.6, 0.8, 1.0, and 1.2 A g^−1^ (based on the mass of sulfur; 201, 173, 145, 116, 94 mAh g^−1^ based on the total mass of electrodes), respectively. By using the WiBS electrolytes, higher capacities (218, 180, 153, 122, and 100 mAh g^−1^ at 0.4, 0.6, 0.8, 1.0, and 1.2 A g^−1^) can be achieved, which can be attributed to the protection of inorganic‐rich SEI layer on the S/AC anode. Such an inorganic‐rich SEI layer may be a result of the decomposition of LiNO_3_ metal salt.^[^ ^33^ ^]^ When deploying MXene interlayer in ASHBs, S/AC/MXene|WiBS|I_2_/CC cells exhibit increased discharge plateau voltages and reduced polarizations, which can also be confirmed by the differential capacity as a function of voltage (dQ/dV) (see details in Figure S21, Supporting Information). Furthermore, S/AC/MXene|WiBS|I_2_/CC cells can demonstrate superior rate performance, delivering 846, 679, 556, and 462 mAh g^−1^ at current densities of 0.4, 0.6, 0.8, and 1.0 A g^−1^ (based on the mass of sulfur; 242, 194, 159, 132 mAh g^−1^ based on the total mass of electrodes), respectively. Even at the current density of 1.2 A g^−1^, full cells can exhibit a capacity of 371 mAh g^−1^ based on the mass of sulfur (106 mAh g^−1^ based on the total mass of electrodes). It should be noted that the short‐chain polysulfides tend to dissolve in aqueous electrolytes, which leads to a relatively lower capacity compared with the theoretical value.^[^ ^34^ ^]^ Figure 4c and Table S2 (Supporting Information) exhibit the capacity contribution from Cl^0^/Cl^−^ and I^+^/I^0^ redox couples under different current densities in the S/AC/MXene|WiBS|I_2_/CC full cell. Obviously, the full cell can deliver a high capacity contributed from Cl^0^/Cl^−^ redox couple (≈50% at 0.4 A g^−1^). The capacity contribution from the Cl^0^/Cl^−^ redox couple decreases with the increase of the current densities, while the capacity contribution from the I^+^/I^0^ redox couple gradually increases. Even at a high current density of 1.2 A g^−1^, Cl^0^/Cl^−^ redox couple can still contribute ≈15% of the total capacity, suggesting the highly efficient Cl^0^/Cl^−^ conversion in ASHBs.

The cycling performances and corresponding voltage profiles of the ASHBs are presented in Figure 4d,e and Figure S22 (Supporting Information). The S/AC|5 m  LiCl|I_2_/CC and S/AC|1 m LiCl–1 m  LiNO_3_|I_2_/CC cells exhibit poor cycling performance (only 26.5% capacity retention after 150 cycles; 33.6% capacity retention after 300 cycles) with low Coulombic efficiencies, implying that the low‐concentrated halide cannot stabilize the I^+^/I^0^ redox reaction nor realize Cl^0^/Cl^−^ redox reaction. By using 20 m  LiCl and WiBS electrolytes, ASHBs deliver improved cycling performance with 53.9% and 58.4% capacity retention after 300 cycles, respectively. This improvement arises from the strong solvation of ions, which immobilizes a large number of water molecules, thereby reducing the amount of free water molecules. This drastically suppresses the disproportionation reaction, preventing rapid capacity degradation. Additionally, substantially increased electrolyte viscosity hinders the diffusion of active species within the liquid phase. Moreover, the formation of high‐quality SEI layers further inhibits the diffusion of active species.^[^ ^8c^ ^]^ Furthermore, superior cycling stability can be achieved by employing the MXene interlayer. The S/AC/MXene|WiBS|I_2_/CC cells can remain at 78.2% capacity after 300 cycles, which demonstrates that the terminations on MXene present strong interaction with polysulfides, thus inhibiting the shuttling of the active species. Noticeably, the capacity fading is primarily attributed to the co‐dissolution of active species on both cathode and anode, with polysulfide dissolution being the dominant factor, as shown in Figure S23 (Supporting Information). The electrochemical impedance spectroscopy (EIS) measurements were performed to investigate the interfacial properties of ASHBs (Figure S24 and Table S3, Supporting Information). The S/AC/MXene|WiBS|I_2_/CC cell shows a reduced interfacial resistance compared with the S/AC||I_2_/CC cells, further suggesting that the reaction kinetics of sulfur can be effectively improved by the formation of stable solid/electrolyte interphase and the introducing of MXene interlayer, which can benefit for the long‐term cycling stability.^[^ ^35^ ^]^

Electrodes with different mass ratios of sulfur and iodine were also evaluated by repeated galvanostatic charging/discharging at a current density of 1.0 A g^−1^ in S/AC/MXene|WiBS|I_2_/CC cell. Figure S25 (Supporting Information) presents the cycling performances and corresponding voltage profiles of various ASHBs with different mass ratios. By using the mass ratio of 1:0.5, the extremely poor cycling stability (30.8% after 150 cycles) may be attributed to the insufficient I^+^ ions, which cannot stabilize the large amount of Cl^0^ at the cathode. With tuning the mass ratio from 1:1 to 1:2 and 1:5, the capacity retention gradually decreases from 78.2% to 53.6% and 42.9%, respectively, which indicates that the excessive I^+^ may aggravate its dissolution and diffusion.

Additionally, the galvanostatic intermittent titration technique (GITT) measurements were carried out to further examine the reaction kinetics of ASHBs. In S/AC|20 m  LiCl|I_2_/CC cells (Figure S26, Supporting Information), the gaps between the voltages of each polarization (red/blue curves) and quasi‐equilibrium (black curve) are approximately in the range of 170–200 mV during the most of charge/discharge process, suggesting the relatively poor reaction kinetics. In contrast, by using optimized electrolyte and MXene interlayer, full cells exhibit lower voltage gaps of 60–95 mV (Figure 4f). Such a small overpotential confirms the fast conversion kinetics in S/AC/MXene|WiBS|I_2_/CC cells. Figure 4g and Table S4 (Supporting Information) compare the energy densities, capacities, and output voltages of different aqueous battery systems based on chalcogen and halogen conversion chemistry. The as‐assembled ASHBs show a superior energy density of 304 Wh kg^−1^_total electrodes_ with an average output voltage of 1.32 V, which significantly surpasses those of the conventional zinc–iodine aqueous batteries.^[^ ^36^ ^]^ Furthermore, ASHBs can also exhibit marvelous flexibility. Both the electrodes (S/AC anode and I_2_/CC cathode) impregnated by WiBS electrolyte can be arbitrarily bent and twisted at any angle. Particularly, the flexible ASHB that is bent to 30° at a fully charged state can maintain the open‐circuit voltage (1.58 V), which demonstrates that the ASHB exhibits a stable electrochemical performance under extreme mechanical forces (Figure 4h). Furthermore, to satisfy the practical application, a bipolar stacking cell has been developed to enhance the output voltage of battery packs. As shown in the insert of Figure 4h, an LED bulb (≈3.0 V) can be powered, which further proves the universality and electrochemical stability of ASHB and enables it as a promising candidate for a power supply.

Although the ASHB systems provide new insight into developing low‐cost energy storage technologies with high energy density, their practical applications still face several challenges. First, while the high‐concentration electrolyte systems can effectively suppress the dissolution and diffusion of polysulfides, they remain fundamentally constrained by the thermodynamic limitations of solid–liquid phase equilibrium, preventing complete immobilization of polysulfides. This can eventually result in active material loss and capacity degradation. Additionally, the excessively high operating potential of the Cl^0^/Cl⁻ redox couple may induce side reactions (such as oxygen evolution), which not only reduces the Coulombic efficiency but also induces continuous degradation of the electrode/electrolyte interface, significantly compromising the cycling stability of batteries. Overcoming these inherent limitations requires coordinated innovations across multiple dimensions, including electrolyte engineering, interface optimization, and the design of novel redox‐active couples.

## 3 Conclusion

In summary, a low‐cost aqueous sulfur‐dual halogen chemistry has been developed by using the WiBS electrolyte, sulfur composite anode, and I_2_/CC cathode, which enables highly efficient energy storage at room temperature. By utilizing the WiBS electrolyte, the Coulomb efficiencies of full cells have been significantly improved due to the restrained side reactions (e.g., electrolyte decomposition and toxic Cl_2_ gas evolution). Furthermore, a co‐optimization strategy for both the cathode/anode has been applied to mitigate the shuttle effects of active species. The elemental iodine has been introduced as a coordinating agent, not only providing extra capacity and supplying I^+^ to stabilize Cl^−^ by forming interhalogen, but also facilitating [ICl_x_]^1−x^ interhalogens oxidation and fixing the Cl^0^ species by forming I^+^─Cl^0^ chemical bonds. Meanwhile, the MXene interlayer on the sulfur anode can effectively confine polysulfides via strong chemical interaction. Therefore, the as‐assembled ASHBs can deliver a capacity of 242 mAh g^−1^_total electrode_ (846 mAh g^−1^_sulfur_) with an output voltage of 1.32 V, thus achieving a high energy density of 304 Wh kg^−1^_total electrodes_. Such ASHBs can also maintain a capacity retention of 78.2% within 300 cycles, demonstrating good cycling stability. These inspiring findings may provide new insights into the high energy density and low‐cost large‐scale energy storage applications.

## Conflict of Interest

The authors declare no conflict of interest.

## Supporting information



Supporting Information

## Data Availability

The data that support the findings of this study are available from the corresponding author upon reasonable request.
