# Supplementary material for: A Highly Reversible Aqueous Sulfur‐Dual‐Halogen Battery Enabled by a Water‐in‐Bisalt Electrolyte
Source: Small. 2025 Apr 17;21(23):2502228. doi: 10.1002/smll.202502228 (PMC12160677; doi:10.1002/smll.202502228)
Supplement: Supplementary file 1 — Supporting Information [file SMLL-21-2502228-s001.docx]

**Supporting Information**

**A highly reversible aqueous sulfur–dual-halogen battery enabled by a water-in-bisalt electrolyte**

*Ronghuan Liang,^1,#^ Yan Wang,^1,#^ Chuanlong Wei,^1^ Xiao Tang,^1,*^ Timing Fang,^1^ Zhezheng Ding,^1^ Qing Wang,^1^ Rui Wang,^1^ Jianjun Song,^2^ Bing Sun,^3,*^ Xiaomin Liu,^1,*^ Guoxiu Wang^3,*^*

^1^ School of Chemistry and Chemical Engineering, Qingdao University, Qingdao, Shandong, 266071, Shandong, P. R. China.

^2^ College of Physics, Qingdao University, Qingdao, Shandong, 266071, Shandong, P. R. China.

^3^ Centre for Clean Energy Technology, School of Mathematical and Physical Sciences, Faculty of Science, University of Technology Sydney, Ultimo, NSW 2007, Australia.

***Corresponding authors:**

E–mail: tangxiao@qdu.edu.cn (X. Tang)

bing.sun@uts.edu.au (B. Sun)

liuxiaomin@qdu.edu.cn (X. Liu)

guoxiu.wang@uts.edu.au (G. Wang)

^#^ These authors contributed equally to this work.

**Methods**

**Materials.** The sulfur powder (S, 99.99%) were purchased from Sigma–Aldrich *LLC*. Lithium chloride (LiCl, 99%), lithium nitrate (LiNO_3_, 99%), and iodine (I_2_, ≥ 99.8%) were purchased from Macklin *Co.*, *Ltd.* Commercial carbon cloth (CC) were purchased from Jiaxing Naco New Materials *Co.*, *Ltd.* The nanoporous activated carbon (AC, YP–80F) was purchased from *Kuraray Co., Ltd.* The titanium carbide aluminum (Ti_3_AlC_2_) precursor was purchased from Xianfeng Nanomaterial Technology *Co., Ltd.*

**Preparation of electrolytes.** The WiBS electrolyte was prepared by dissolving 15 mol kg^–1^ (m) LiCl and 7 m LiNO_3_ in deionized water. Additionally, 5 m and 20 m LiCl electrolytes were prepared for comparison. All the electrolytes were continuously bubbled with N_2_ gas flow for several minutes before usage.

**Synthesis of the Ti_3_C_2_T_x_ MXene.** The Ti_3_C_2_T_x_ MXene can be obtained by a typical LiF/HCl etching process according to the previous report.^[1]^ Typically, the 1 g Ti_3_AlC_2_ powder was slowly added into the LiF/HCl solution at 35°C for 24 h with stirring. Subsequently, the obtained multilayered Ti_3_C_2_T_x_ suspension was repeatedly washed with deionized water and centrifuged (10 min under 3000 r/min) until the pH of the supernatant was higher than 5.

**The preparation of S/AC/MXene composite anode.** As for the preparation of S/AC composite anode, the sulfur powder and conductive activated carbon powder were ground in a mass ratio of 4:6 with heating at 155℃ for 12 h.^[2]^ Subsequently, the S/AC anode can be prepared by compressing the mixture of S/AC powder, conductive carbon black, and 5 wt% polyvinylidene fluoride aqueous solution with a mass ratio of 8:1:1 on the titanium mesh (200 mesh, 18 μm). Adoption of titanium mesh current collectors not only presents corrosion during cycling, but also reduces the strain on the electrodes to avoid the cracks formation.^[2]^ The average mass loading of sulfur is ~2.68 mg cm^–2^. The S/AC/MXene composite anode can be prepared by uniformly coating Ti_3_C_2_T_x_ MXene suspension on the S/AC anode (~0.3 mg cm^–2^) and dried under vacuum condition at 40 ℃ for 6 h.

**The preparation of I_2_/CC freestanding composite cathode.** The iodine/carbon cloth (I_2_/CC) cathode can be prepared by a “solution–adsorption” method.^[3]^ Initially, a piece of porous commercial carbon cloth (CC) was repeatedly cleaned by anhydrous ethanol, diluted hydrochloric acid, and distilled water. Subsequently, CC was put into distilled water with a certain amount of iodine powder with slow stirring at 60 °C for 24 h. The elemental iodine can absorb into the CC until the solution turns clear. All the CC and I_2_/CC cathode were dried at 40 °C in vacuum oven for 12 h. The areal mass loading of iodine is ~3.08 mg cm^–2^.

**Characterizations.** The morphology and microstructure of S/AC anode, MXene, and I_2_/CC cathode were characterized by applying the field emission scanning electron microscope (FE–SEM, JSM–78OOF, JEOL). The corresponding elements EDX mapping were detected by energy dispersive spectrometer (EDS, JSM–7800F, JEOL). Thermogravimetric analysis (TGA) was performed on a TGA/DSC1 system under Ar_2_ atmosphere with a heating rate of 10 ℃ min^–1^ from room temperature to 600°C. Raman spectra was tested by Renishaw inVia Raman spectrometer system with a laser of 585 nm wavelength. As for the *in situ* Raman measurements, the S/AC/MXene|WiBS|I_2_/CC full cell was assembled in a home–made glass cell. Ultraviolet–visible spectrometer (UV–vis, UV–2600, SHIMADZU) measurement was performed ranging from 200 to 600 nm. It should be noted that all the analyzed samples were obtained by immersing cycled I_2_/CC cathode into the WiBS electrolyte. The cycled I_2_/CC cathodes were disassembled from full cells at different charge/discharge states. The ^1^H nuclear magnetic resonance spectroscopy was measured in Nuclear magnetic resonance (NMR, JNM–ECZ600R/S1, JEOL) with dimethyl sulfoxide (DMSO) as the reference, which was used to determine intermolecular interactions. The conversion route of I and Cl elements in the composite cathode was carried out by the X–ray photoelectron spectroscopy (XPS, ESCALAB Xi+, Thermo Fisher) with a limiting vacuum of 5 × 10^–10^ mbar. The C 1*s* (284.8 eV) was calibrated as a reference before analyzing the valence evolution of all elements.

**The assembly of aqueous batteries.** The Nafion membranes (N117) were utilized as separators, which were purchased from DuPont China Holding *Co., Ltd*. The pre–treated membranes were achieved by a typical method. Firstly, the membranes were immersed in 5 wt% H_2_O_2_ (Reagent *Co., Ltd*) at 80 ℃ for 1 h to remove organic impurities, and was immersed in deionized water for 0.5 h. Subsequently, the membranes were soaked in 1 M H_2_SO_4_ (Reagent *Co., Ltd*) at 80 ℃ for 1 h to remove metallic impurities and to acidify the sulfonic sites,^[4]^ which was then immersed in deionized water for 0.5 h. Finally, membranes were immersed in 1 m LiNO_3_ aqueous solution for 2 h, which could change the H^+^ conductive to Li^+^ conductive Nafion membranes.^[5]^ The thickness of membrane was 180 μm.

The aqueous S/AC||CC batteries were assembled in a Swagelok cell in the air condition by using the S/AC anode, CC cathode, separator, and different aqueous electrolytes. The electrode areas of the S/AC anode and CC cathode were 0.4 × 0.5 cm^2^. Before assembling, the CC cathode was initially rinsed repeatedly with anhydrous ethanol, hydrochloric acid, and deionized water to remove impurities. As for the assembly of aqueous sulfur–dual halogen batteries (ASDBs), the full cells were assembled in a Swagelok cell by using the S/AC anode (or S/AC/MXene anode), I_2_/CC cathode, and separator. The mass ratio of sulfur to iodine was set as 1:1. The electrode areas of the S/AC anode and I_2_/CC cathode were 0.4 × 0.5 cm^2^ and 0.4 × 0.4 cm^2^, respectively.

**Electrochemical measurements.** Cyclic voltammetry (CV) and chronopotentiometry measurements of three-electrode configurations were performed by using an electrochemical workstation (CHI 660E, Chenhua) with titanium mesh as the working electrode, saturated silver/silver chloride (Ag/AgCl) as the reference electrode, and platinum wire as the counter electrode. Electrochemical impedance spectra (EIS) of full cells were carried out on CHI 660E electrochemical workstation with a frequency range of 10^–2^~10^5^ Hz and a disturbance amplitude of 5 mV. The CV curves of full cells were tested at a scan rate of 0.2 mV s^–1^ on CHI 660E electrochemical workstation. Cycling performance, rate capability, and galvanostatic intermittent titration technique (GITT) measurements of full cells were performed on a Neware battery test system (CT–4008Tn) at room temperature. The voltage range of full cells were set as 0.6~1.8 V. Before the electrochemical tests, the as–assembled full cells were first activated by cycling at 0.4 A g**^–^**^1^_sulfur_ for 5 cycles at room temperature. The GITT measurements were performed by a series of GCD pulses at 0.4 A g**^–^**^1^_sulfur_ for 10 min with alternating for 30 min rest periods until voltage reached 0.6 or 1.8 V. The capacities were calculated based on the mass of sulfur or total electrodes (S/AC and I_2_). The energy density calculation is conducted based on the following equation:

E = C × V_mid_  (S1)

where E is energy density (Wh Kg^–1^), C is capacity (mAh g^–1^) based on the total mass of active species, and V_mid_ (V) is mid–value voltage.

**Computational details.** Molecular dynamics (MD) simulations were conducted to study the solvation structures and scrutinize the free water content of different aqueous electrolytes. The MD simulations were run by using LAMMPS.^[6]^ The systems are setup initially by using PACKMOL and Moltemplate (http://www.moltemplate.org/).^[7]^ The properties of H_2_O are assessed with SPC/E parameters. The force–fields parameters and partial charges of Li^+^, Cl^–^, and NO_3_^–^ are taken from previous publications.^[8]^ The compositions of simulated electrolytes are given in **Table S1**. The density functional theory (DFT)–D3 calculation was carried out using the Gaussian 09 software. The structural optimization was performed at M06–2X/def2–TZVP level. Vibrational frequencies were calculated at the same level of theory to ensure that the optimized structures have no imaginary frequency as the global minima. The Gibbs free energy changes (Δ*G*) were calculated by:

Δ*G* = *G*_P_ – *G*_R_ (S2)

where *G*_R_ is the total Gibbs free energy of reactants and *G*_P_ is the total Gibbs free energy of the products. The Gibbs free energies were the correction of single–point energies, which were calculated at M06–2X/def2–TZVP level.


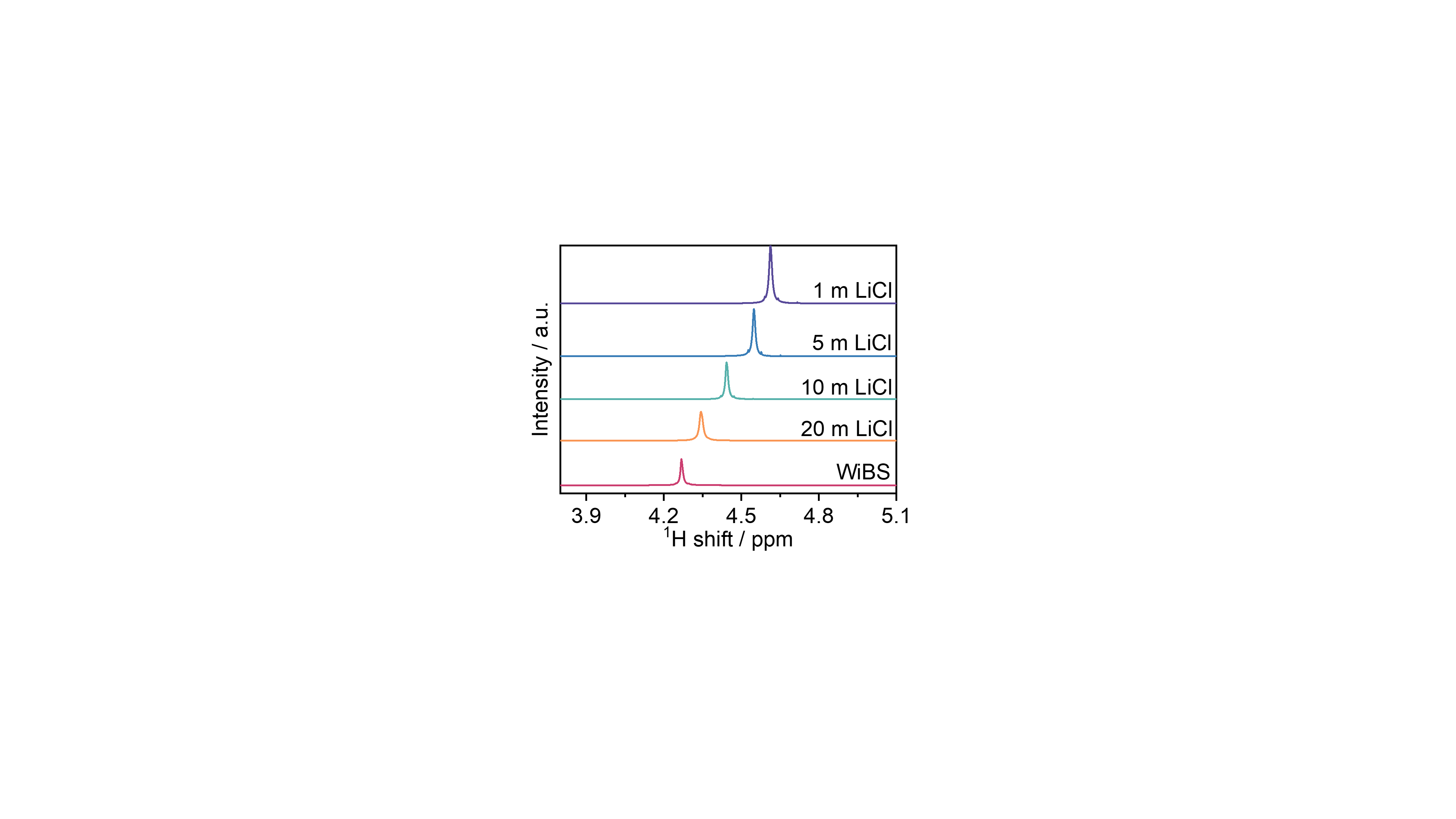


**Figure S1**. The ^1^H NMR spectra of H_2_O for different electrolytes. The ^1^H chemical shifts in 1 m, 5 m, 10 m, 20 m LiCl, and WiBS electrolytes were 4.61, 4.55, 4.44, 4.34, 4.27 ppm, respectively.


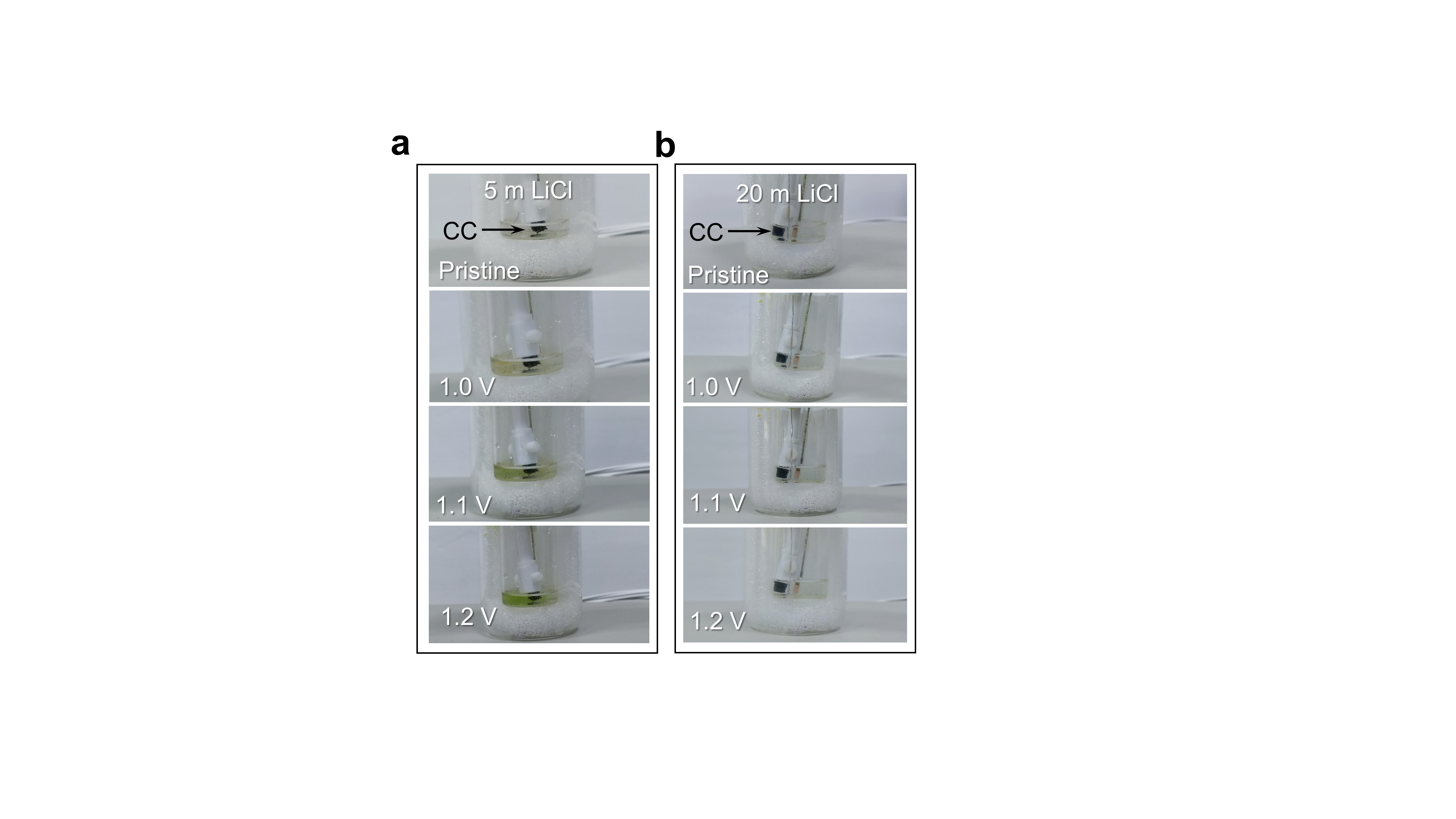


**Figure S2**. The color changing of the (a) 5 m and (b) 20 m LiCl aqueous electrolytes at different potentials. Carbon cloth were used as working electrode.


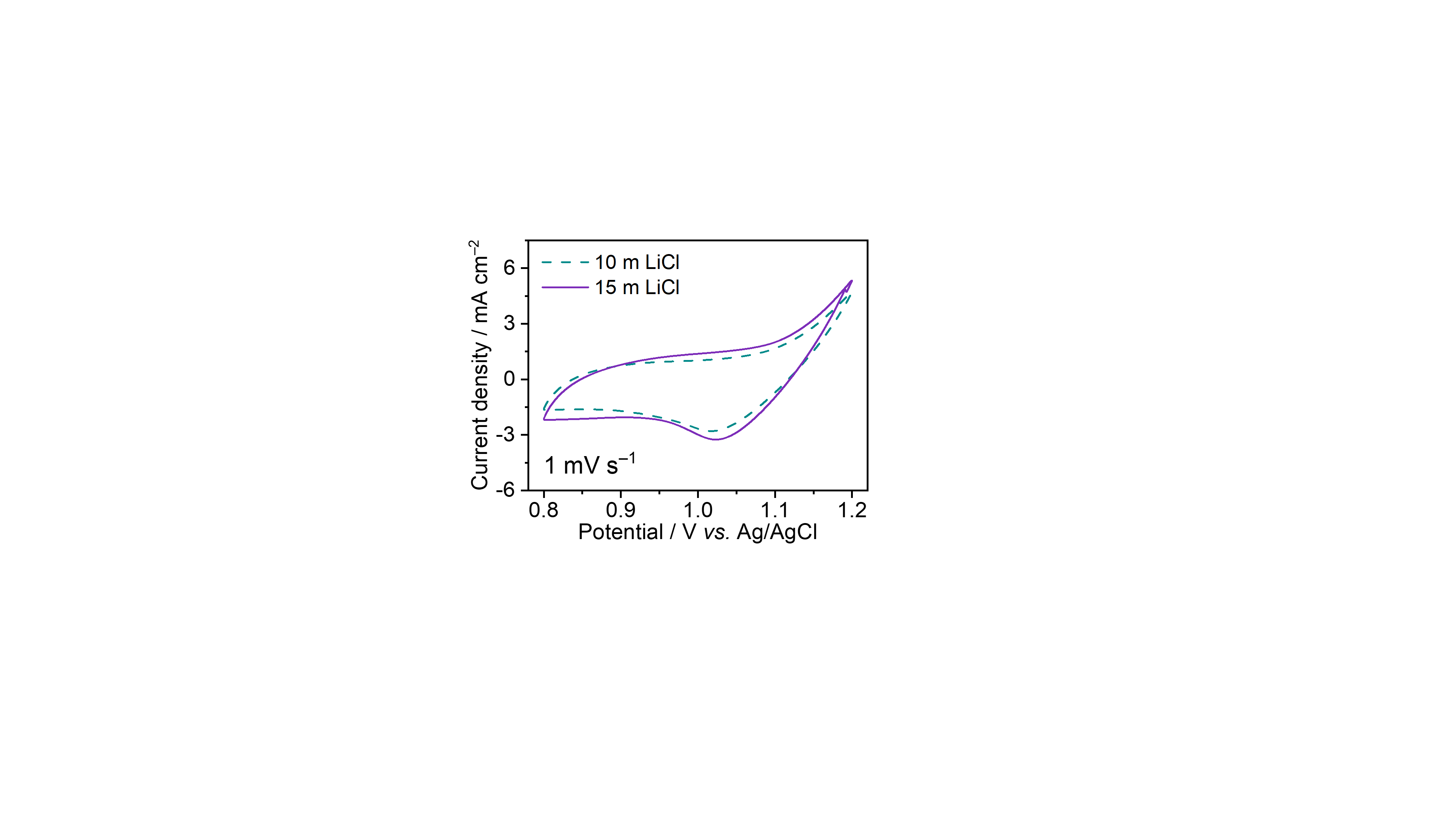


**Figure S3**. The CV curves of bare CC electrode in 10 m and 15 m LiCl electrolytes at a scan rate of 1 mV s**^–^**^1^.


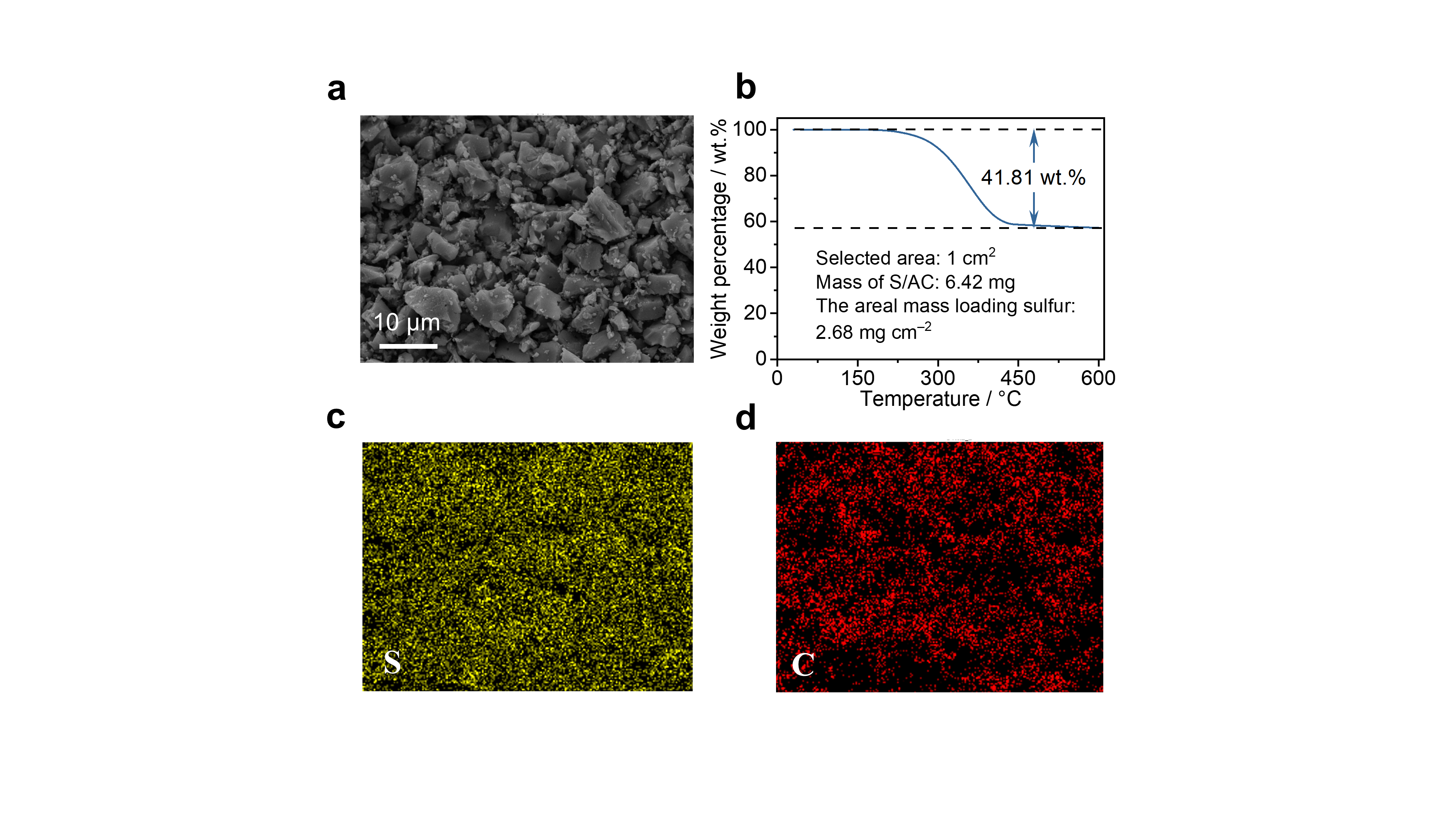


**Figure S4**. (a) SEM image of S/AC composites anode. (b) The thermogravimetry analysis (TGA) curve of S/AC anode under Ar_2_ flow. (c–d) EDX mapping of S and C elements.


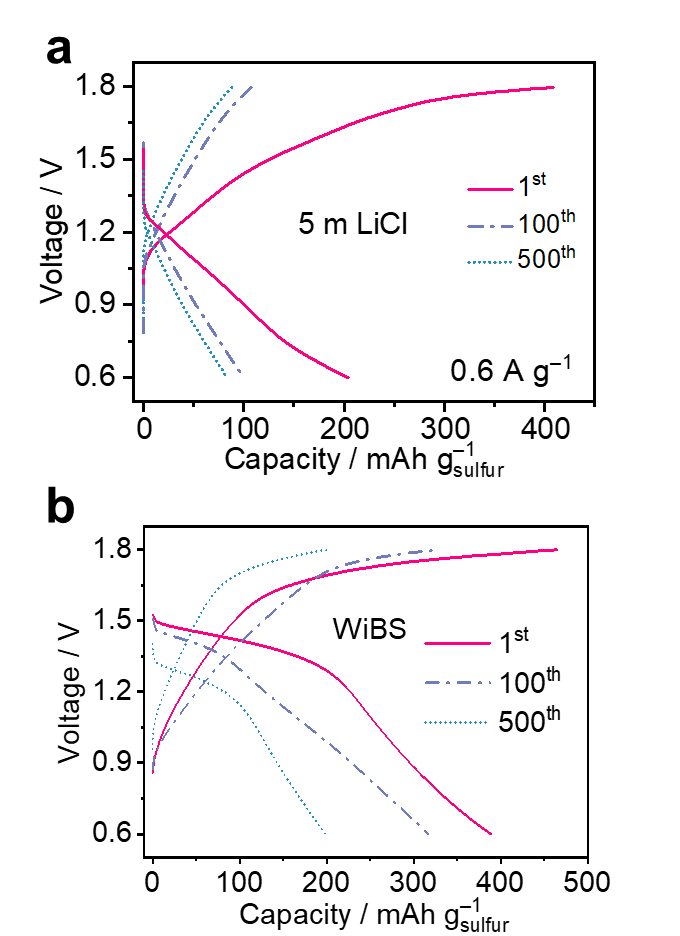


**Figure S5**. The voltage profiles of (a) S/AC|5 m LiCl|CC and (b) S/AC|WiBS|CC cells during 500 cycles at 0.6 A g^–1^_sulfur_. The S/AC|WiBS|CC cells can deliver a capacity of 389 mAh g^–1^ (based on the mass of sulfur) with the output plateau voltage of 1.3V.


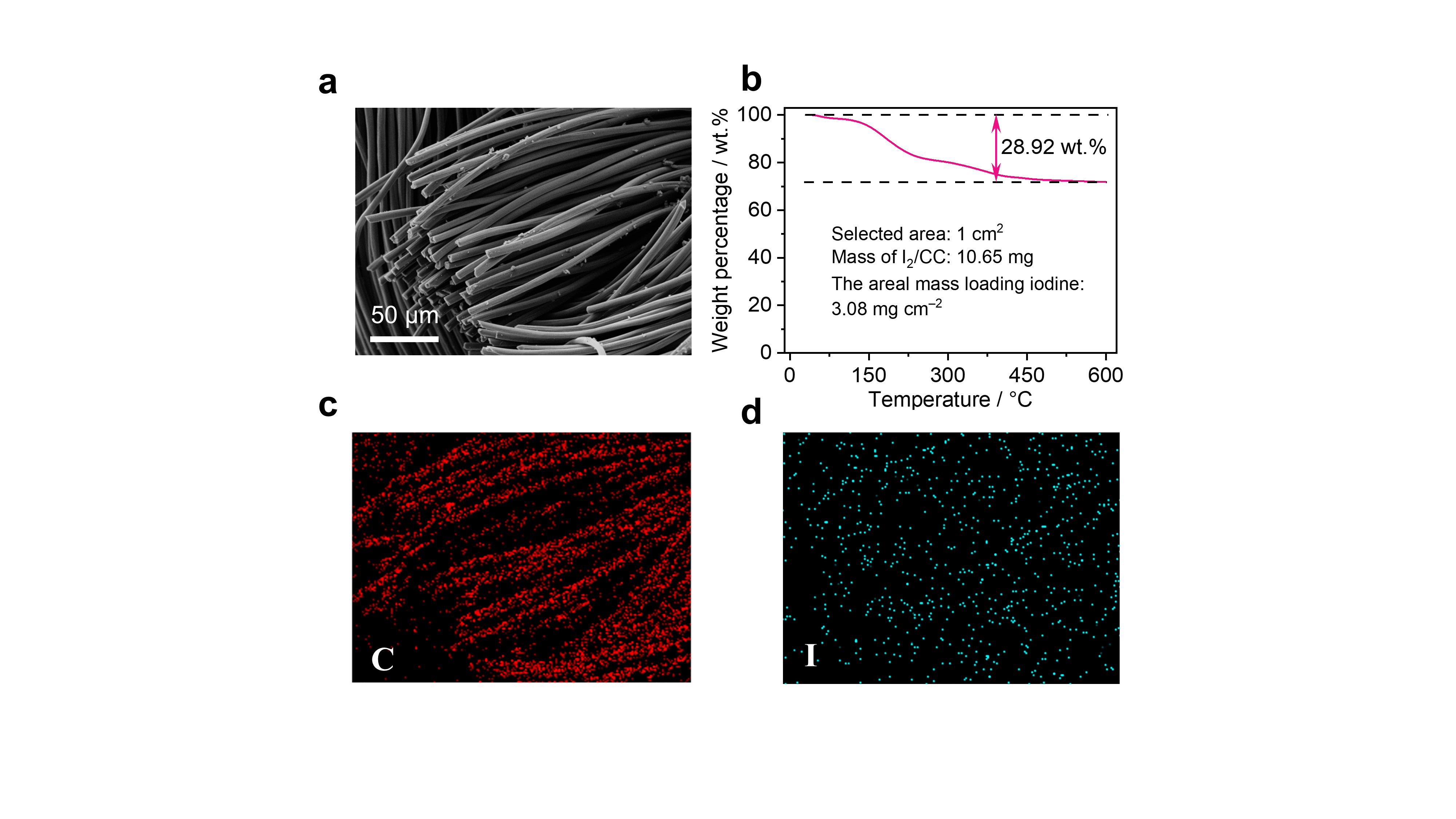


**Figure S6**. (a) SEM image of I_2_/CC composites anode. (b) The TGA curve of I_2_/CC cathode under Ar_2_ flow. (c–d) EDX mapping of C and I elements, respectively.


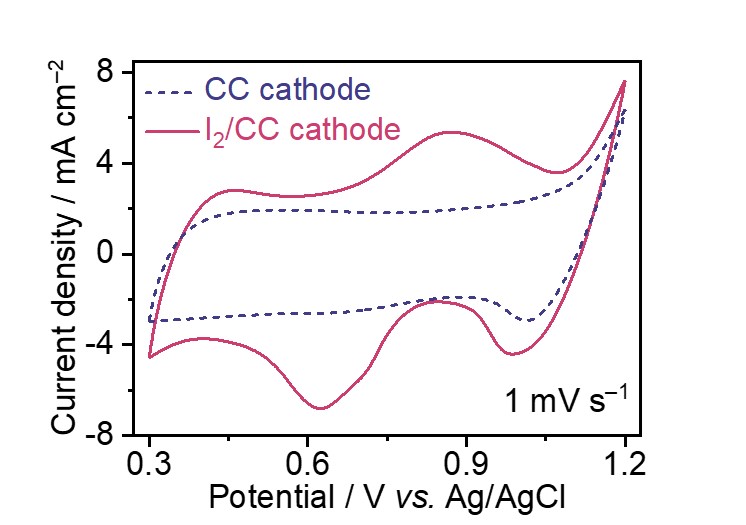


**Figure S7.** CV curves of carbon cloth and I_2_/CC cathode in WiBS electrolyte at a scan rate of 1 mV s^–1^.


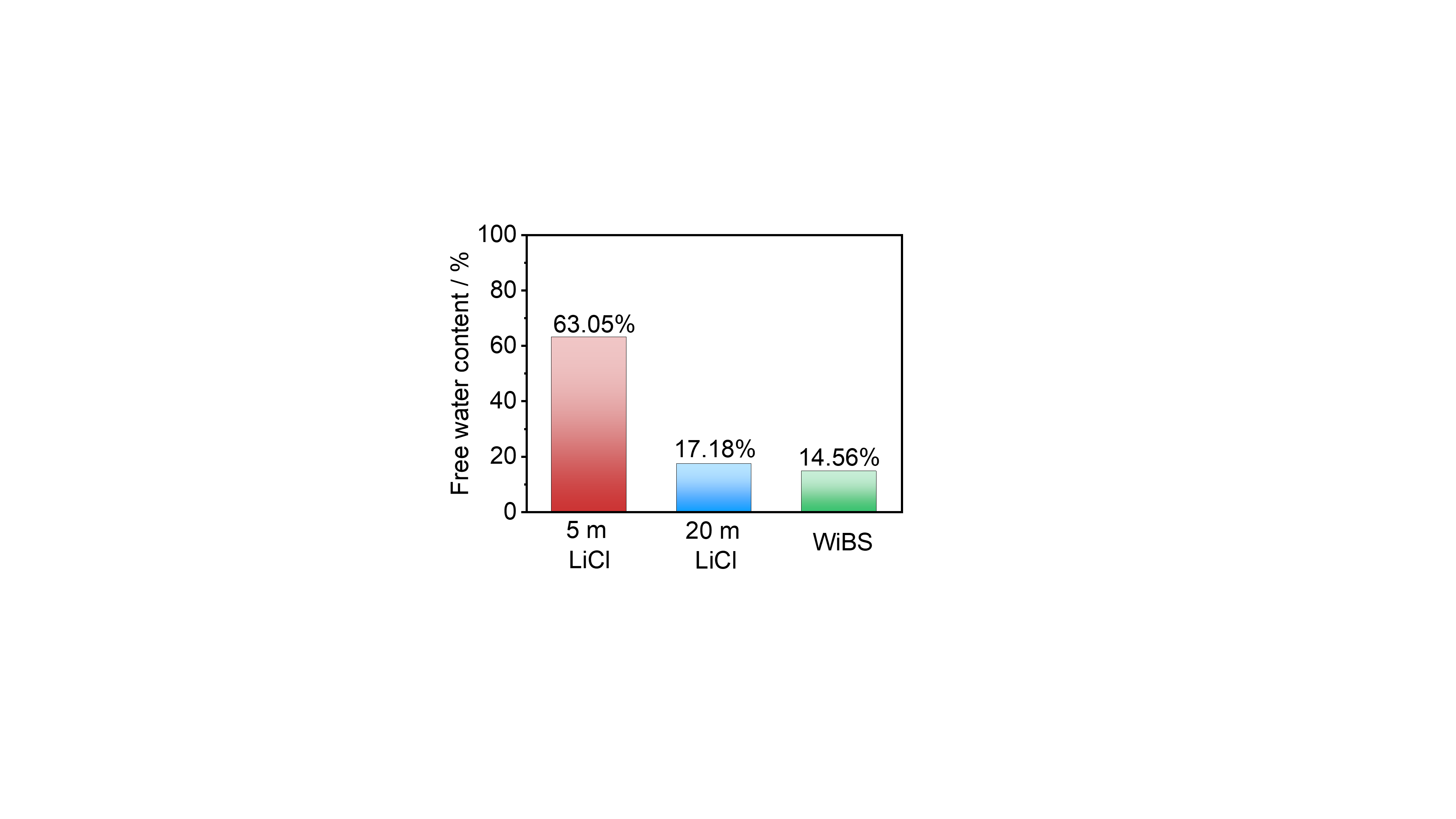


**Figure S8**. The free water content in various aqueous solutions.


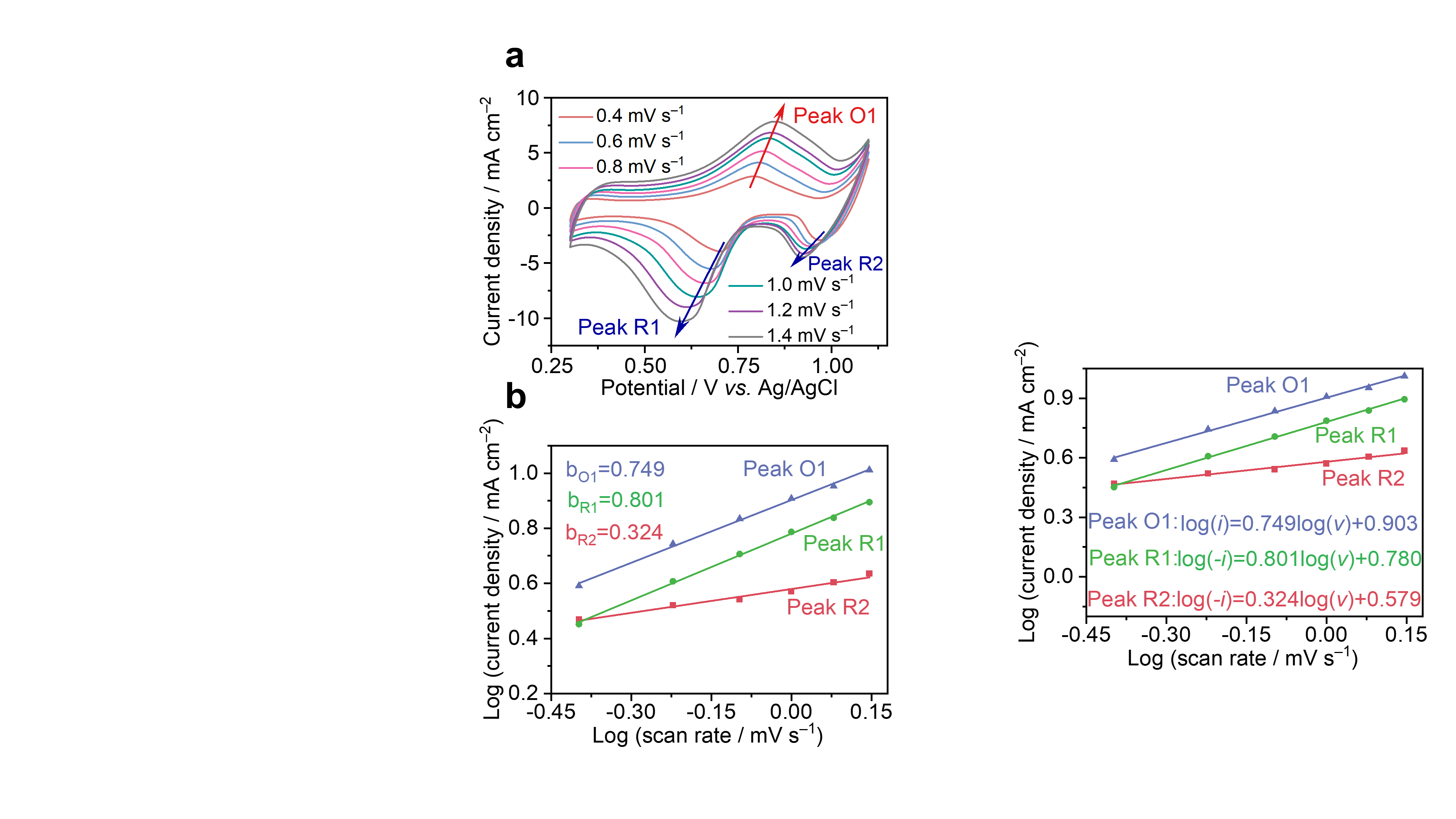


**Figure S9**. (a) The CV curves of the I_2_/CC cathode collected at scan rates of 0.4, 0.6, 0.8, 1.0, 1.2, and 1.4 mV s^–1^. (b) Plot of log (peak current density) as function of log (scan rate) obtained from the oxidation peak in **Figure S9a**.

With the increase of scanning rates, the two pairs of wide redox peaks show an low tendency of shifting (0.70/0.79 V and 0.97/1.10 V at 0.4 mV s^–1^ to 0.59/0.85 V and 0.92/1.10 V at 1.4 mV s^–1^), demonstrating the good reversibility of the continuous two–electron reactions in the I_2_/CC cathode. Furthermore, according to Dunn’s method,^[9]^ the mode of charge storage in I_2_/CC cathode was investigated, which can be illustrated by the following equations:

*i* = a*ν*^b^ (S3)

log(*i*) = blog(*ν*) + log(a) (S4)

Where the *i* is current densities, *ν* is scan rates, a and b are adjustable parameters. In general, when b value is close to 0.5, the capacity is contributed from the diffusion–controlled process; while when b value is close to 1, the capacity is dominated by the capacitive behaviors. In **Figure S9b**, the b value of the oxidation peak 1 and reduction peak 1 corresponding to the I^+^/I^0^ reaction are calculated to be about 0.749 and 0.801, respectively, indicating that the capacity is determined by both the diffusion–controlled and capacitive behaviors. Noticeably, the b value of the reduction peak 2 is calculated to 0.324, featuring the conversion reactions with diffusion determined behavior, which is consistent with the reported work.^[10]^


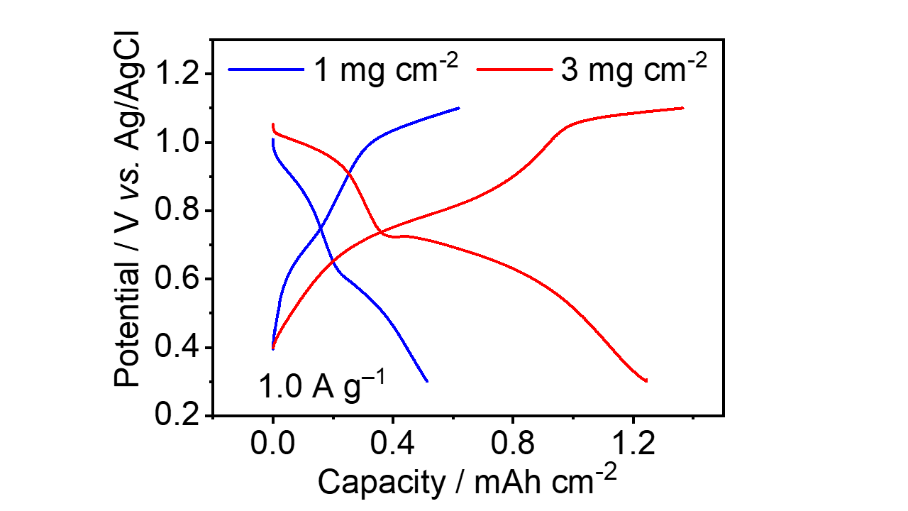


**Figure S10**. The voltage profiles of I₂/CC cathodes with iodine mass loadings of 1 mg cm⁻^2^ and 3 mg cm⁻^2^ in the WiBS electrolyte at a current density of 1.0 A g⁻^1^_iodine_, within the potential range of 0.3~1.1 V (*vs.* Ag/AgCl).


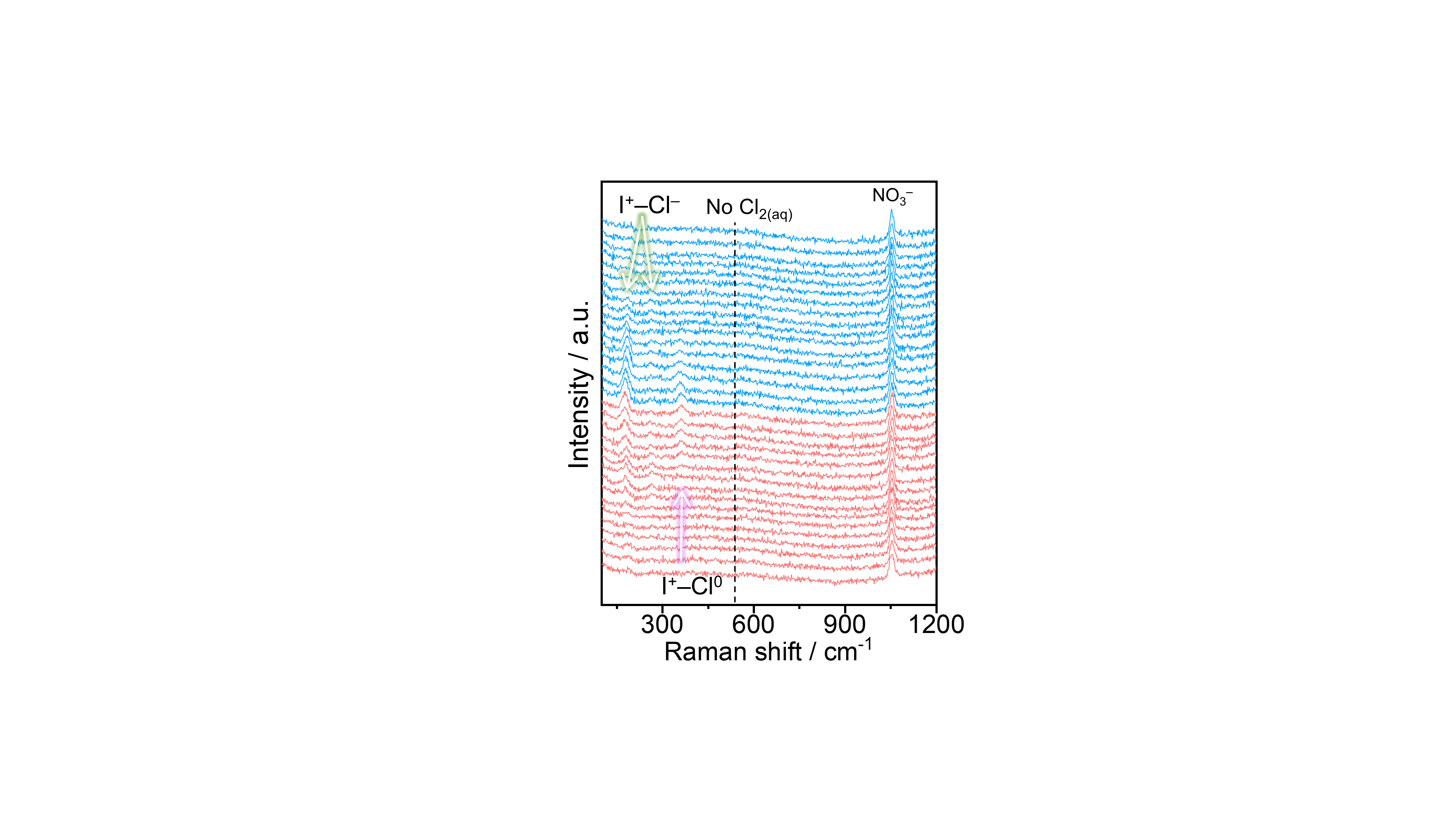


**Figure S11.** *In* *situ* Raman spectra of the I_2_/CC cathode in the S/AC|WiBS|I_2_/CC glass cells during cycling at 0.4 A g^−1^ with the voltage interval of 0.06 V. The red curves and blue curves are collected during the charge and discharge processes, respectively.

The peak appeared at ~1050 cm^–1^ can be assigned to the nitrate anions (NO_3_^–^) in the WiBS electrolyte, which is consistent with the previous reported works.^[11]^ Noticeably, there is no obvious peaks related to the formation of hydrated chlorine (Cl_2_), confirming the stable fixation of Cl^0^ by I^+^ species.


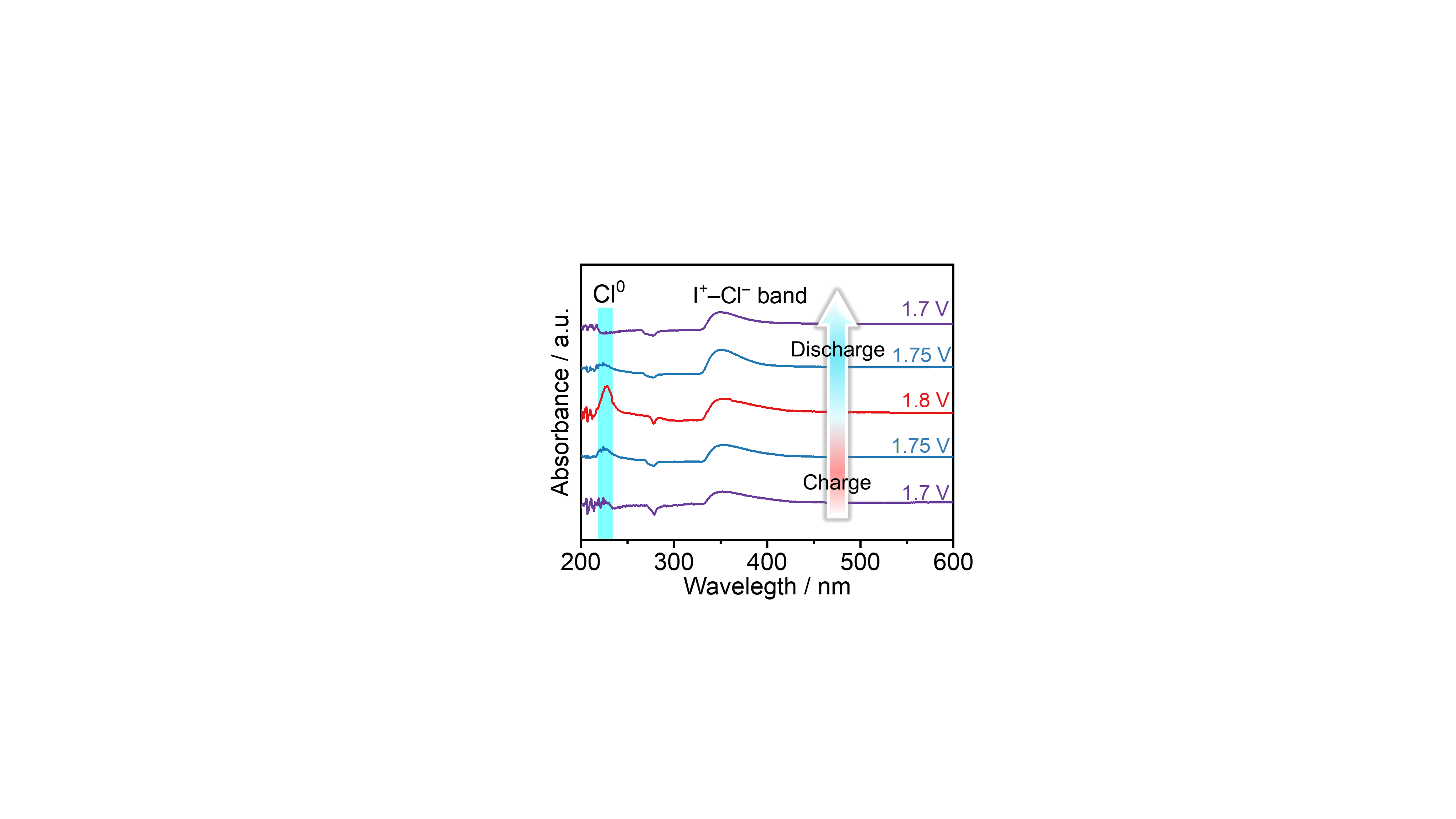


**Figure S12.** The UV–vis spectra of the WiBS electrolytes at different charge and discharge states.

**Figure S12** shows the UV–vis spectra of WiBS electrolyte with immersing the I_2_/CC cathode at different charge/discharge states. By charging I_2_/CC cathode 1.8 V, a wide hump at ~349 nm can be attributed to the formation of [ICl_x_]^1–x^ interhalogens,^[12]^ while a prominent absorption peak appears at ~227 nm, which is related to the generation of Cl^0^ species. This reveals the oxidation process from Cl^−^ to Cl^0^ in interhalogens. Noticeably, peaks of Cl_2_ (~322 cm^–1^) and ClO**^–^** (~284 cm^–1^) are not presented, which means that Cl atoms are preferentially coordinated with iodine rather than spontaneously forming Cl_2_ gas, therefore effectively suppressing the gas evolution and improving the reversibility of chlorine–based redox reaction.


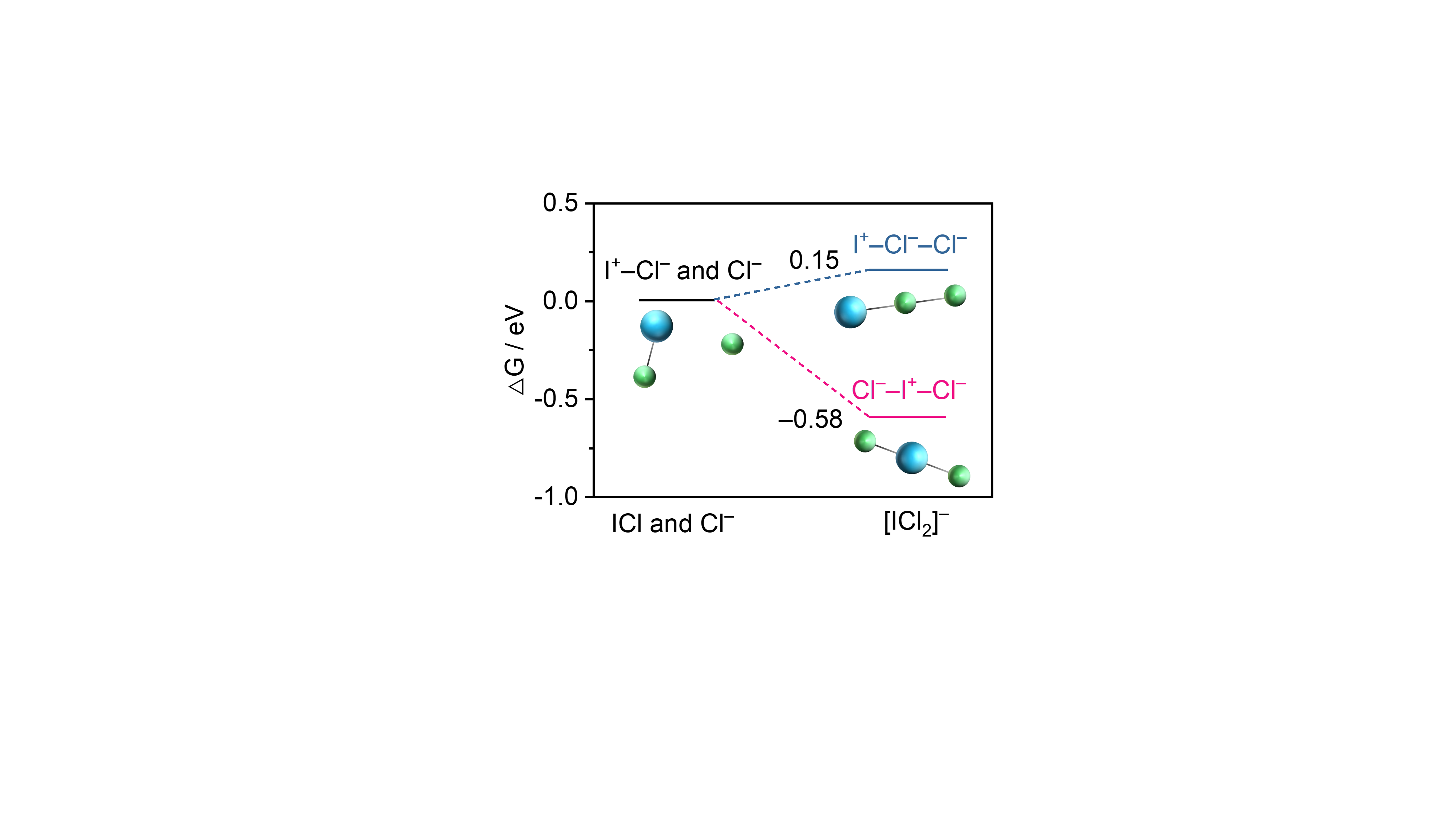


**Figure S13.** The free energy difference between ICl and Cl^–^ with [ICl_2_]^–^. Blue and green represent iodine and chlorine atoms, respectively.


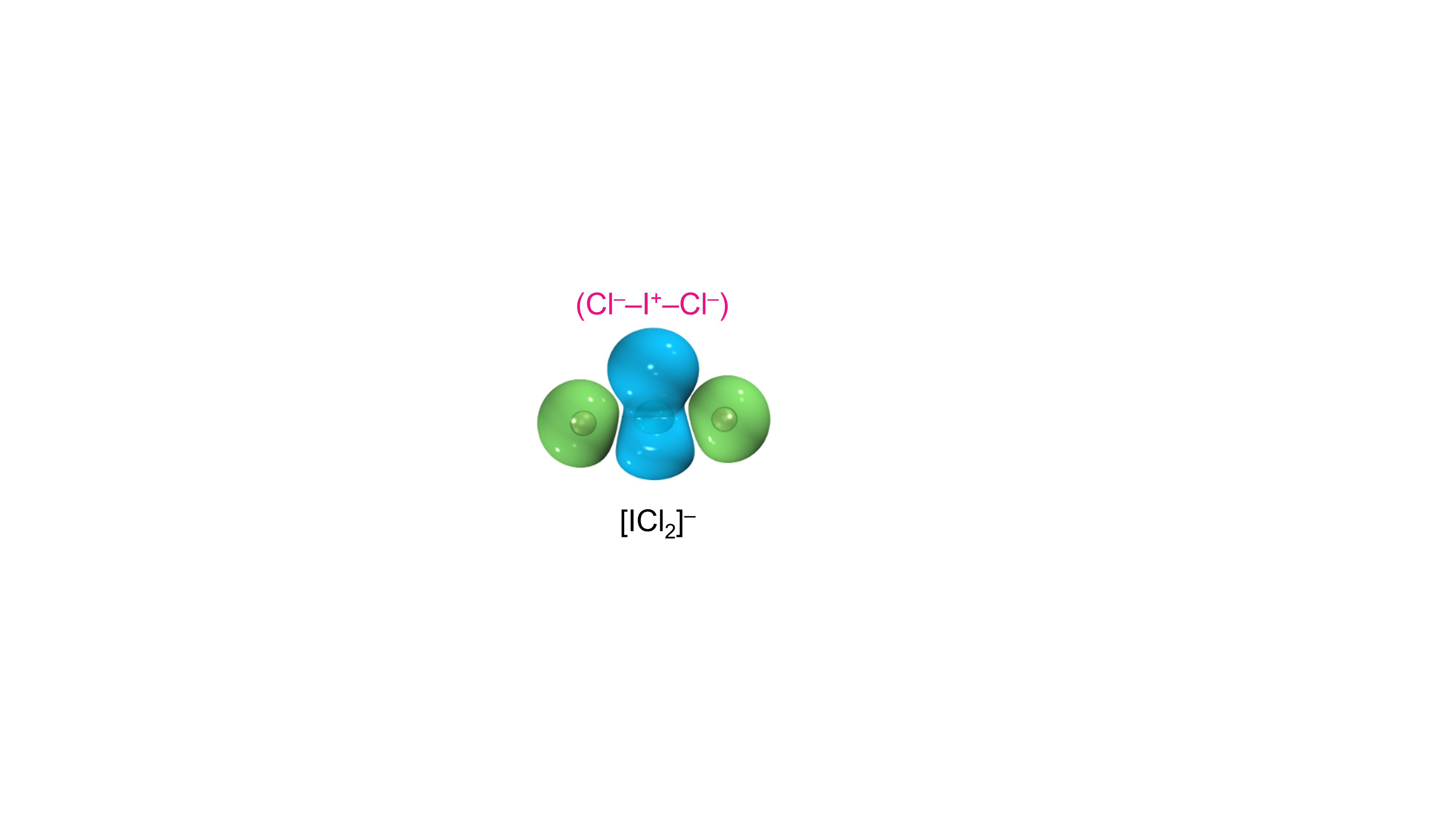


**Figure S14.** The difference in charge density of the [ICl_2_]^–^ with Cl–I–Cl conformation.


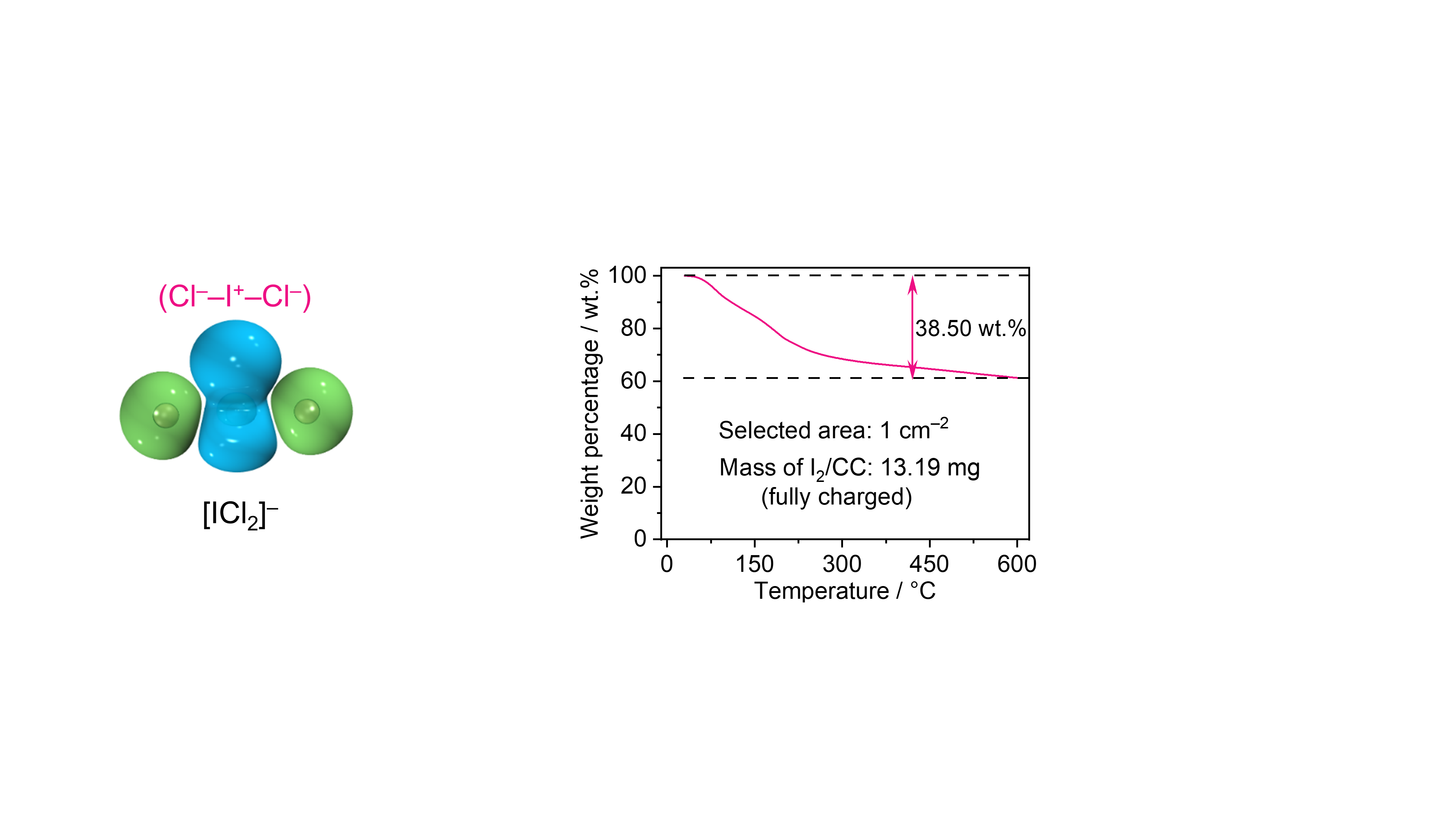


**Figure S15.** The TGA curve of I_2_/CC cathode at fully charged state under Ar_2_ flow.

As shown in **Figure S15**, the weight loss percentage for active species in the fully charged I_2_/CC cathode is ~38.50 wt% when the temperature reached 600°C. The weight loss for active species can be calculated to be 13.19 mg× 38.50% = 5.08 mg. According to **Figure S7b**, the areal mass loading iodine in initial I_2_/CC cathode is ~3.08 mg cm^–2^. Therefore, the weight loss for active species has increased from 3.08 to 5.08 mg, which indicates the formation of interhalogens based on the interhalogen coordinating chemistry (I^+^–Cl^–^ and I^+^–Cl^0^). Thereby, the molar ratio of Cl to I can be calculated as follows:

$$\frac{\text{(5.08–3.08)}}{\text{35.5}}\text{ : }\frac{\text{3.08}}{\text{126.9}}\text{= 2.32 : 1 (S5)}$$

Consequently, the molar ratio of Cl to I is 2.32: 1, which means ~2.32 Cl atoms are coordinated with one I atoms. This confirms that [ICl_2_]^0^ or [ICl_3_]^–^ can be considered as the final products as a consequence of the coordinating effects between I^+^ and Cl^0^ species. This is also consistent with the previously reported results.^[10]^


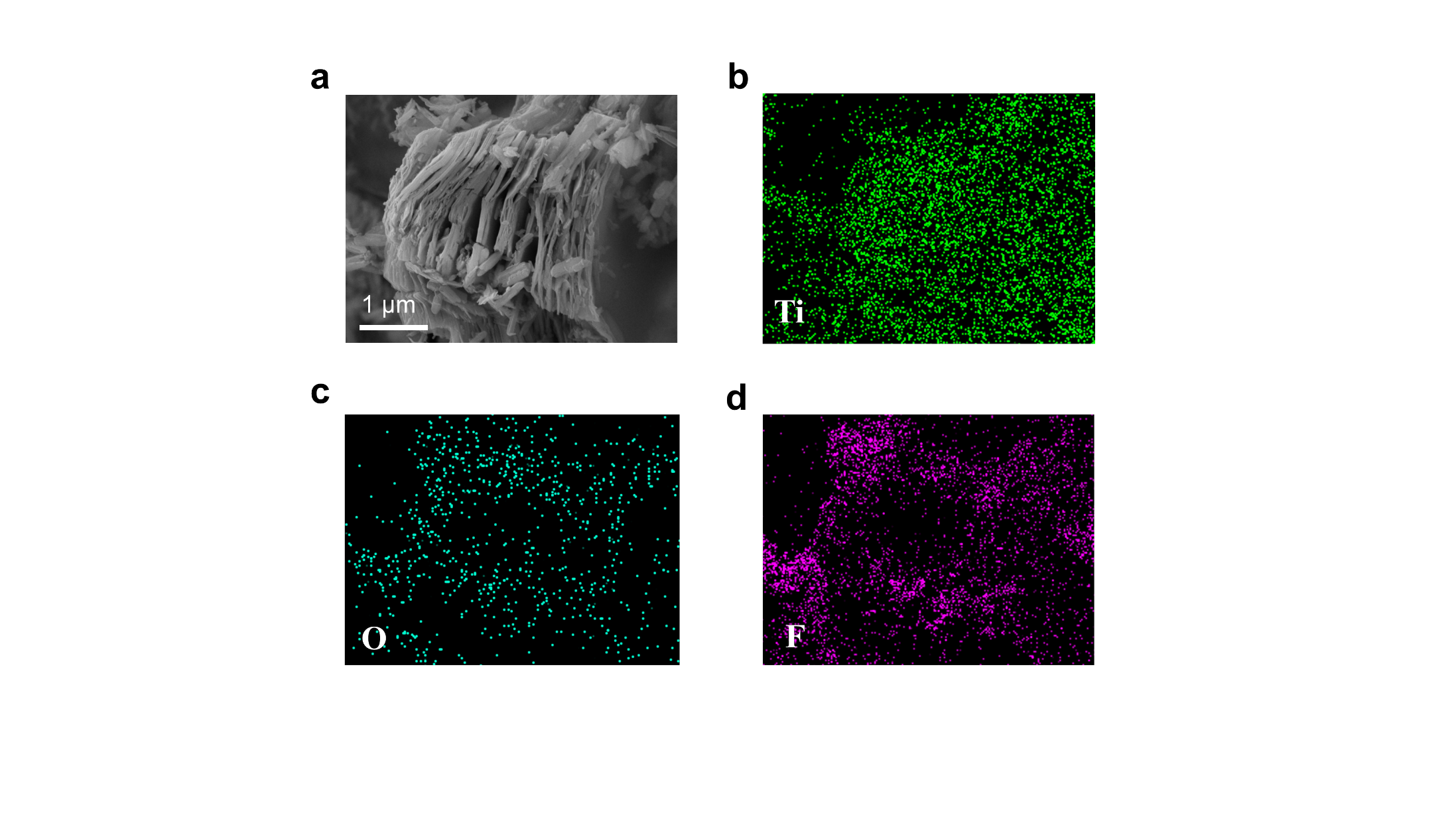


**Figure S16**. (a) SEM image of MXene materials. (b–d) The EDX mapping of Ti, O, and F elements, respectively.


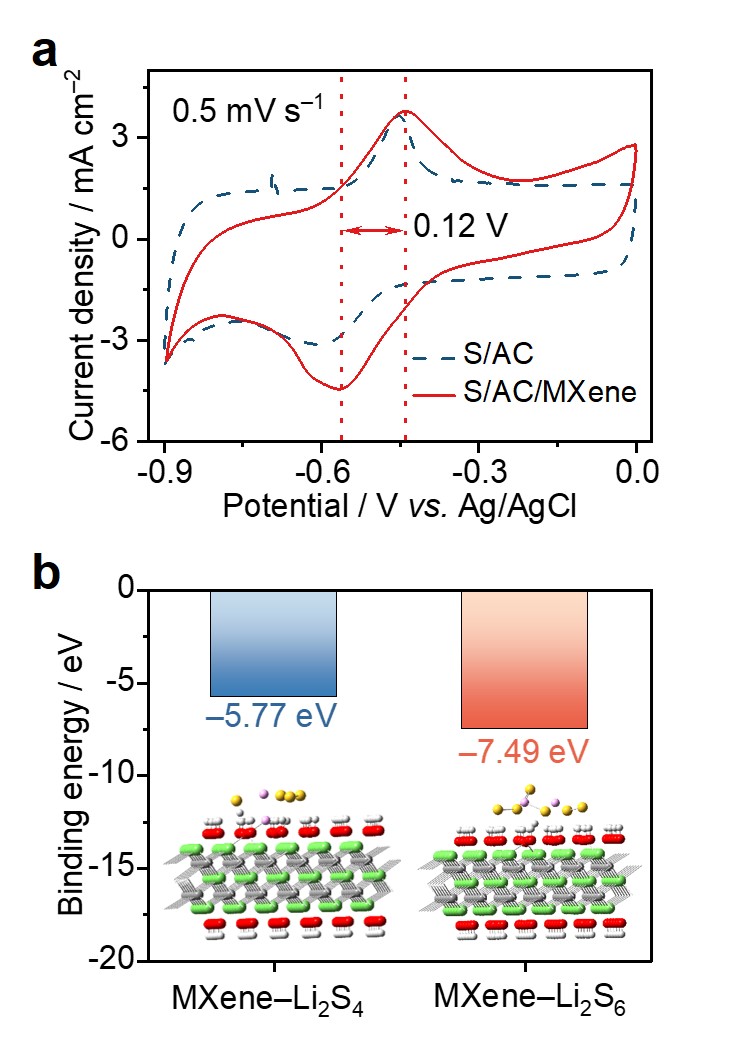


**Figure S17.** (a) CV curves of S/AC and S/AC/MXene anode in WiBS electrolyte at a scan rate of 0.5 mV s^–1^. (b) The comparison of binding energies of MXene toward Li_2_S_4_ and Li_2_S_6_. The insets are the molecular conformation of MXene with Li_2_S_4_ and Li_2_S_6_.


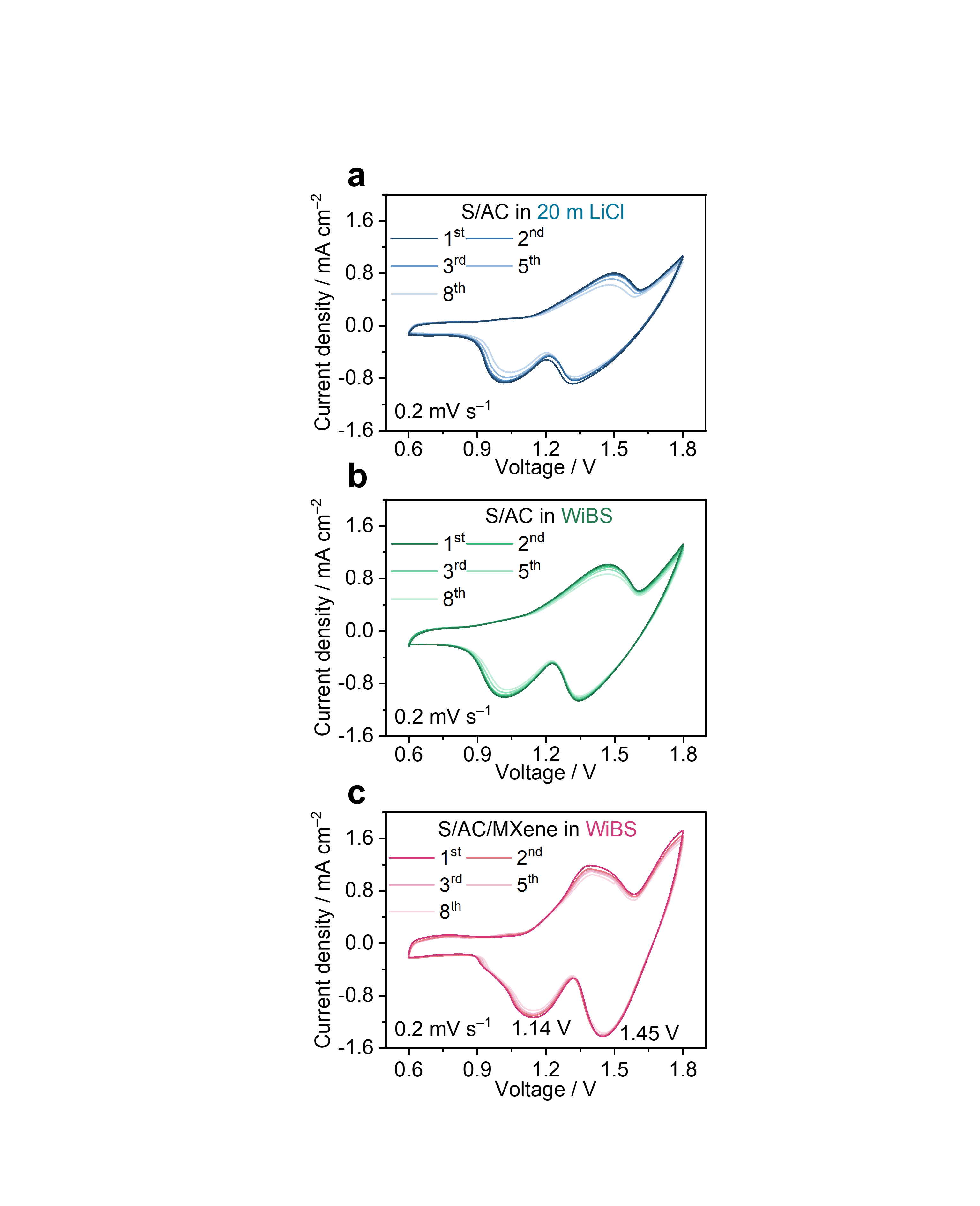


**Figure S18.** (a–c) The comparison of CV curves for different ASDBs at a scan rate of 0.2 mV s^–1^.


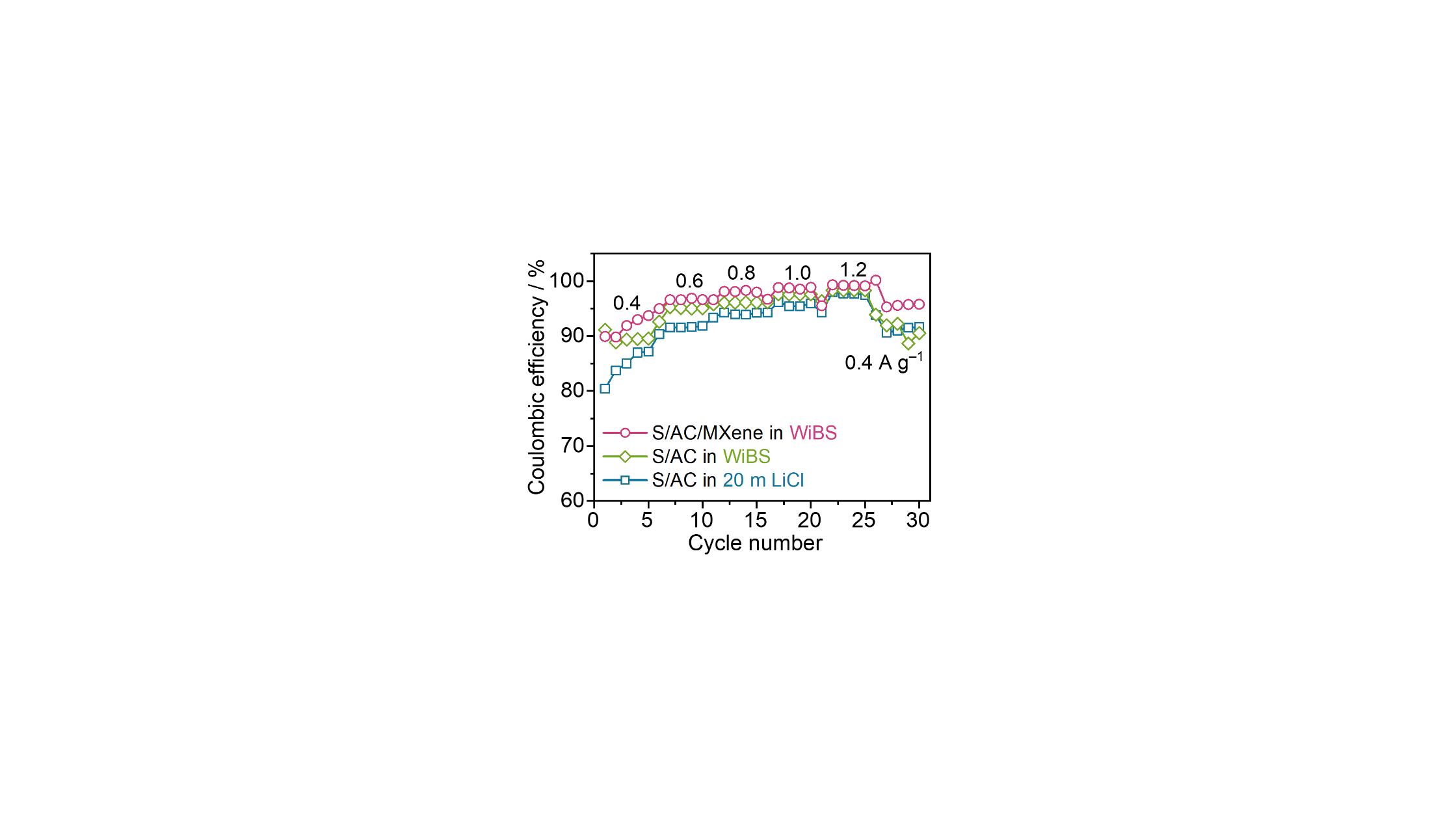


**Figure S19.** The Coulombic efficiencies of different ASDBs at different current densities.


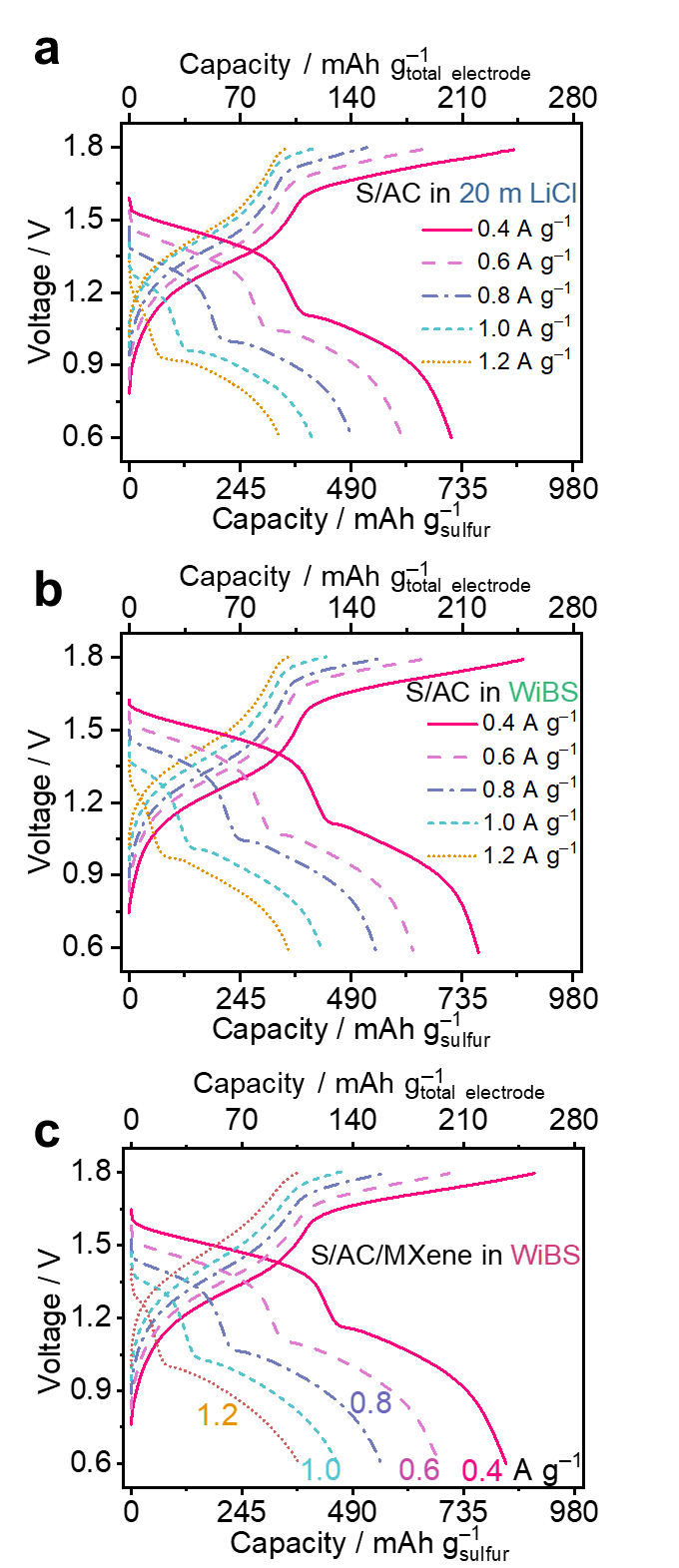


**Figure S20.** The voltage profiles of the S/AC||I_2_/CC full cells with (a) 20 m LiCl and (b) WiBS electrolytes.


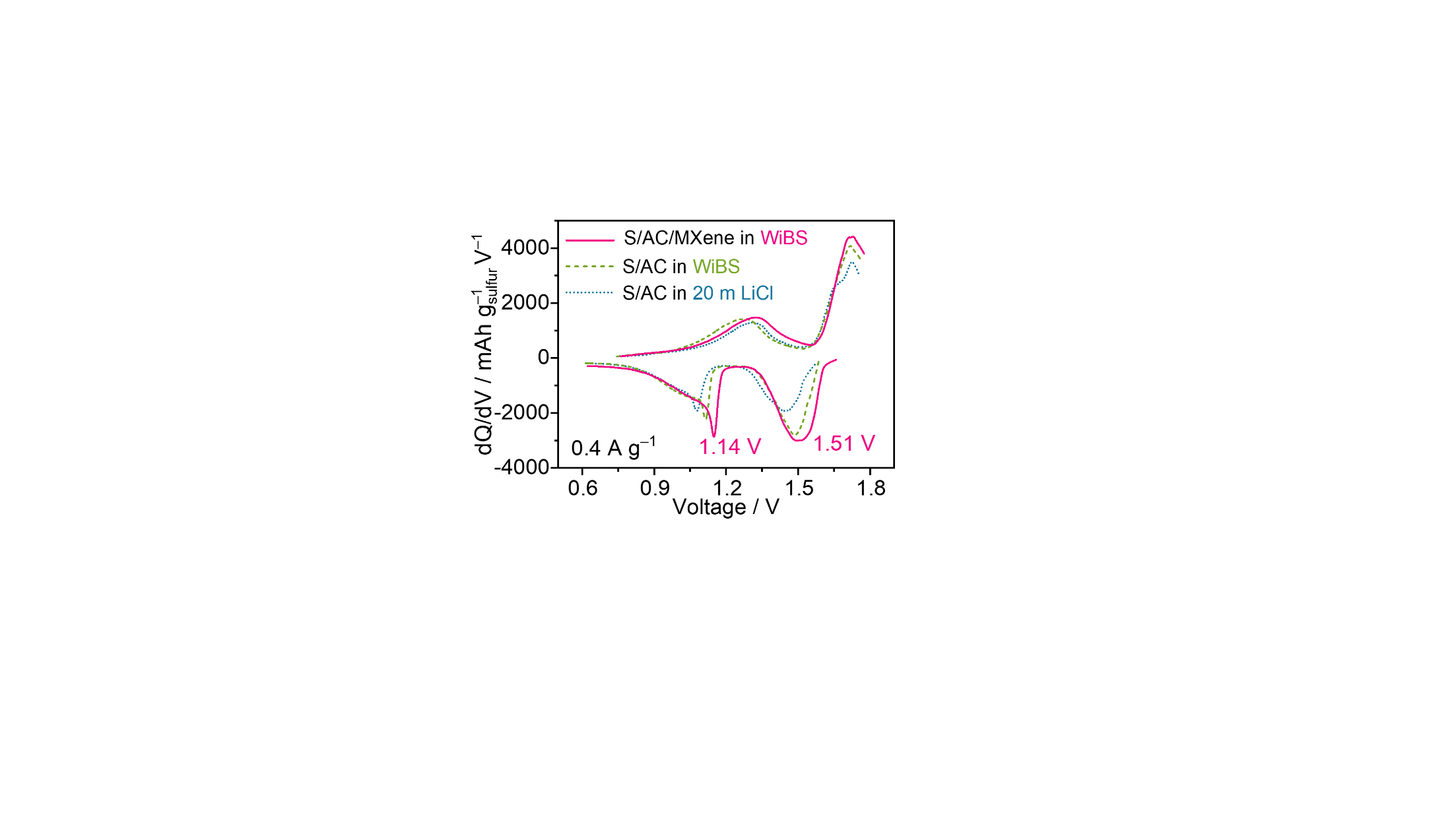


**Figure S21.** The dQ/dV plots of the voltage profiles of different ASDBs at 0.4 A g^–1^.

As shown in **Figure S21**, the differential capacity as function of voltage (dQ/dV) was plotted to elucidate the kinetics evolution of different ASDBs. There are two pairs of redox peaks in the dQ/dV profiles, which can be assigned to the reaction of I^+^/I^0^ and Cl^0^/Cl^–^. Noticeably, the S/AC/MXene|WiBS|I_2_/CC full cells show the higher peak intensities with a smaller polarization, indicating its superior fast reaction kinetics.

**
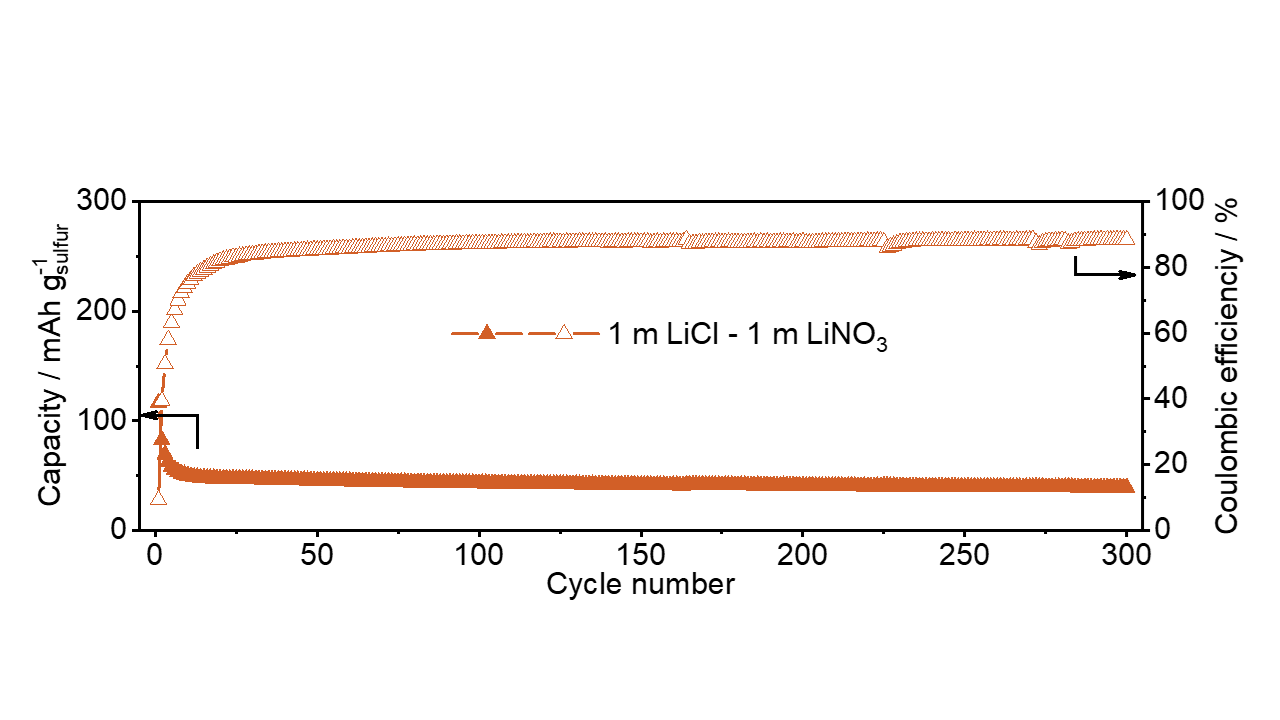
**

**Figure S22.** The cycling performances and corresponding Coulombic efficiencies of S/AC |1 m LiCl–1 m LiNO_3_ |I_2_/CC full cell at 1 A g⁻^1^. The mass ratio of sulfur to iodine was set as 1:1.


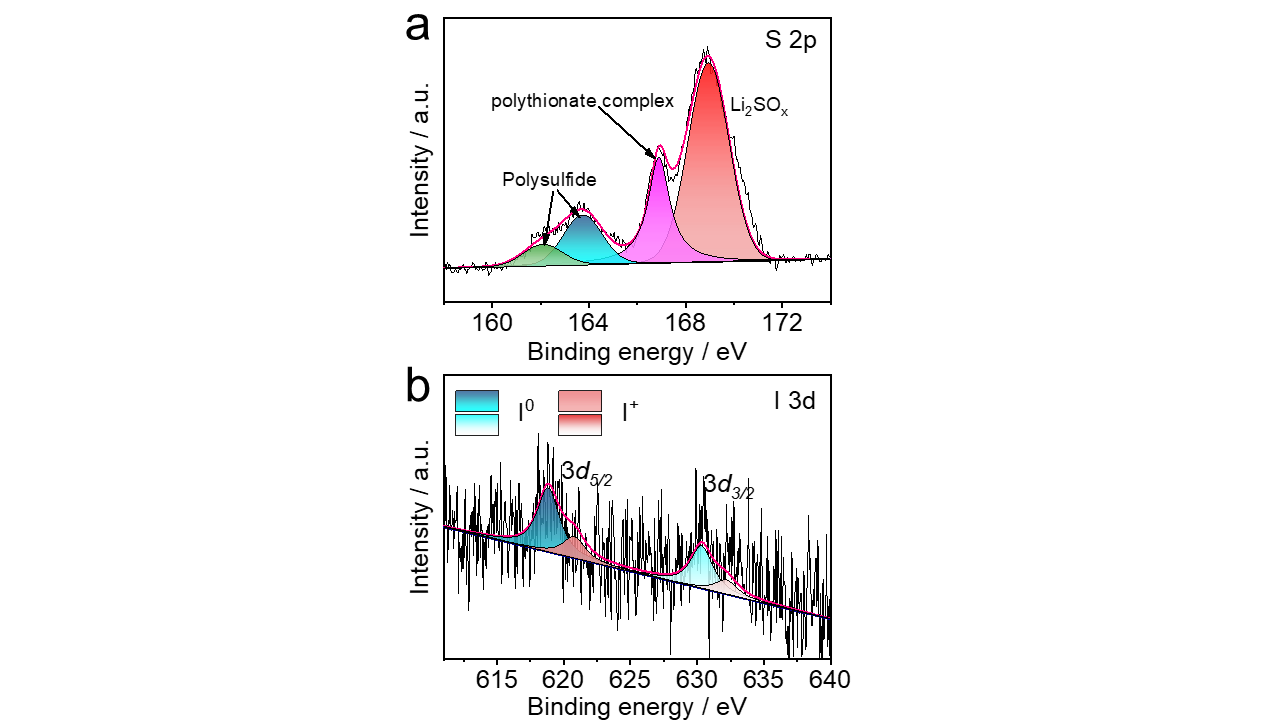


**Figure S23.** (a) S *2p* and (b) I *3d* XPS of glass fiber separator disassembled from a cycled full cell.

We have assembled an ASHB with a glass fiber separator. We performed XPS measurements on the glass fiber separator disassembled from the cycled ASHB. As shown in S *2p* spectrum (**Figure S23a**), the peaks at 162.1 eV and 163.75 eV can be assigned to the polysulfides, while two peaks at 166.9 eV and 168.95 eV can be attributed to polythionate complex and lithium sulfates, respectively.^[13]^ The presence of lithium sulfates (SO_3_^2–^ and SO_4_^2–^) is because the Li^+^ in Li_2_S_4_ tend to be substituted by H^+^ in weak acidic environmental medium to form the by–product HS^–^, which can be further oxidized to SO_3_^2–^ and SO_4_^2–^ due to the trace amount of dissolved oxygen.^[14]^ In the I *3d* spectrum (**Figure S23b**), weak signals of I^0^ and I⁺ were detected at 618.8/630.3 eV and 620.8/632.2 eV, respectively.^[12]^ Additionally, according to our previous work, the WiBS electrolyte possess a broad electrochemical stability window (~2.41 V)^[15]^, which could satisfy the requirement of the as–developed ASHBs. These results indicate that capacity degradation originates from the co-dissolution of active species on both cathode and anode, with polysulfides dissolution being the dominant factor.

**
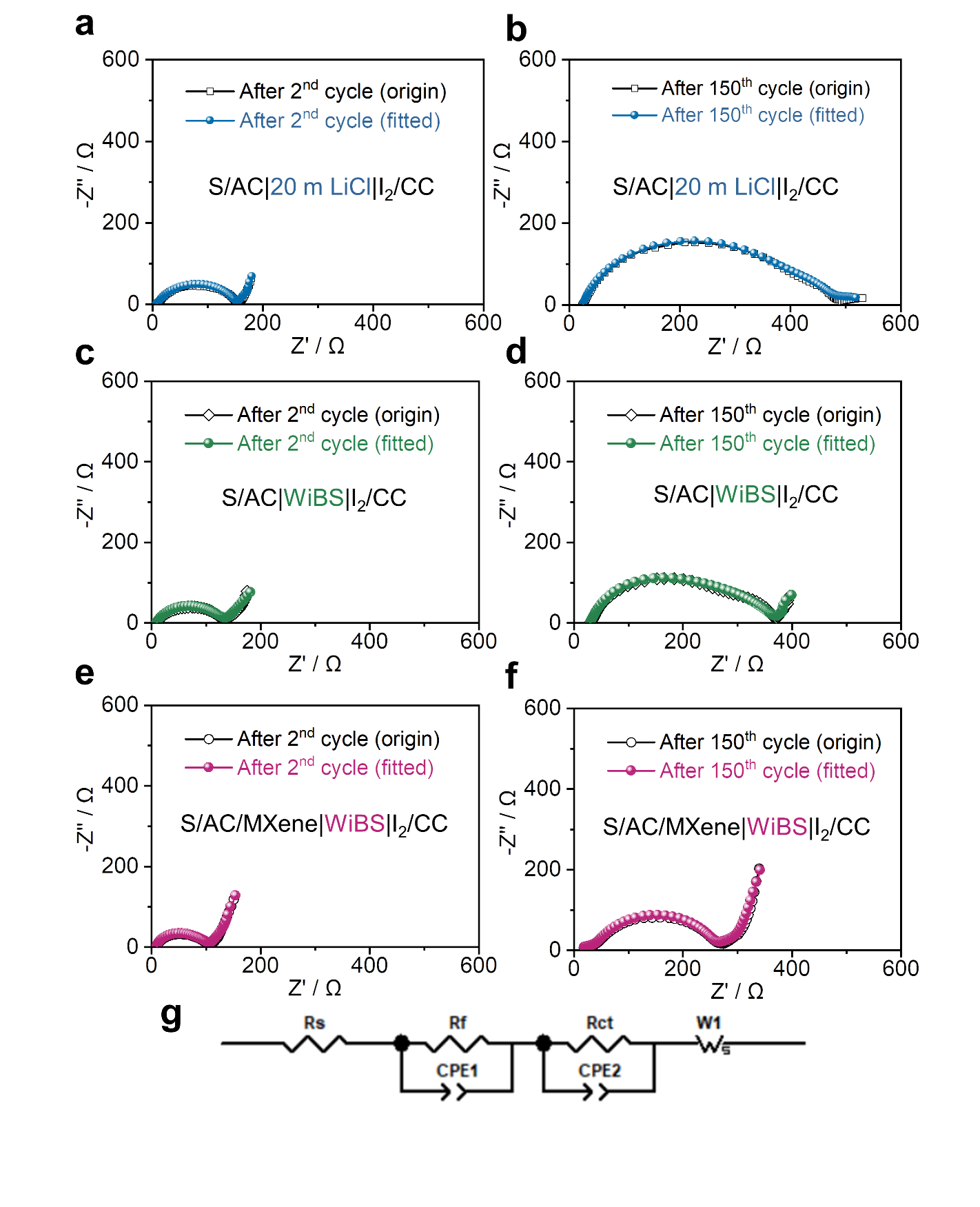
**

**Figure S24.** (a–f) The EISs and fitted EIS curves of the S/AC|20 m LiCl|I_2_/CC, S/AC|WiBS|I_2_/CC, and S/AC/MXene|WiBS|I_2_/CC full cells after 2^nd^ and 150^th^ cycles. (g) The equivalent circuit for fitted EIS curves.

As shown in **Figure S24a–f**, a semicircle can be observed in the Nyquist diagram and fitted into two parts, representing the solid electrolyte interphase resistance (*R_f_*) related to the diffusion behavior of Li^+^ in SEI layers and the charge–transfer resistance (*R_ct_*) between electrolyte and electrodes. The equivalent circuit is shown in **Figure S24g**. According to the fitted results shown in **Table S3**, the R_f_ rapid increases from 24.2 Ω to 325.8 Ω during 2^nd^ to 150^th^ cycles in S/AC|20 m LiCl|I_2_/CC cell, indicating the sluggish conversion kinetics caused by the shuttle effect in batteries. In contrast, the R_f_ merely rises from 21.0 Ω to 220.7 Ω and 39.8 Ω to 120.4 Ω in S/AC|WiBS|I_2_/CC and S/AC/MXene|WiBS|I_2_/CC cells, respectively. These suggests the charge transfer and ionic diffusion has been improved in optimized ASDBs.

**Figure S25.** The cycling performances and corresponding Coulombic efficiencies of ASDBs with different mass ratios of sulfur to iodine (1:0.5, 1:1, 1:2, and 1:5) at a current density of 1 A g^–1^_sulfur_.


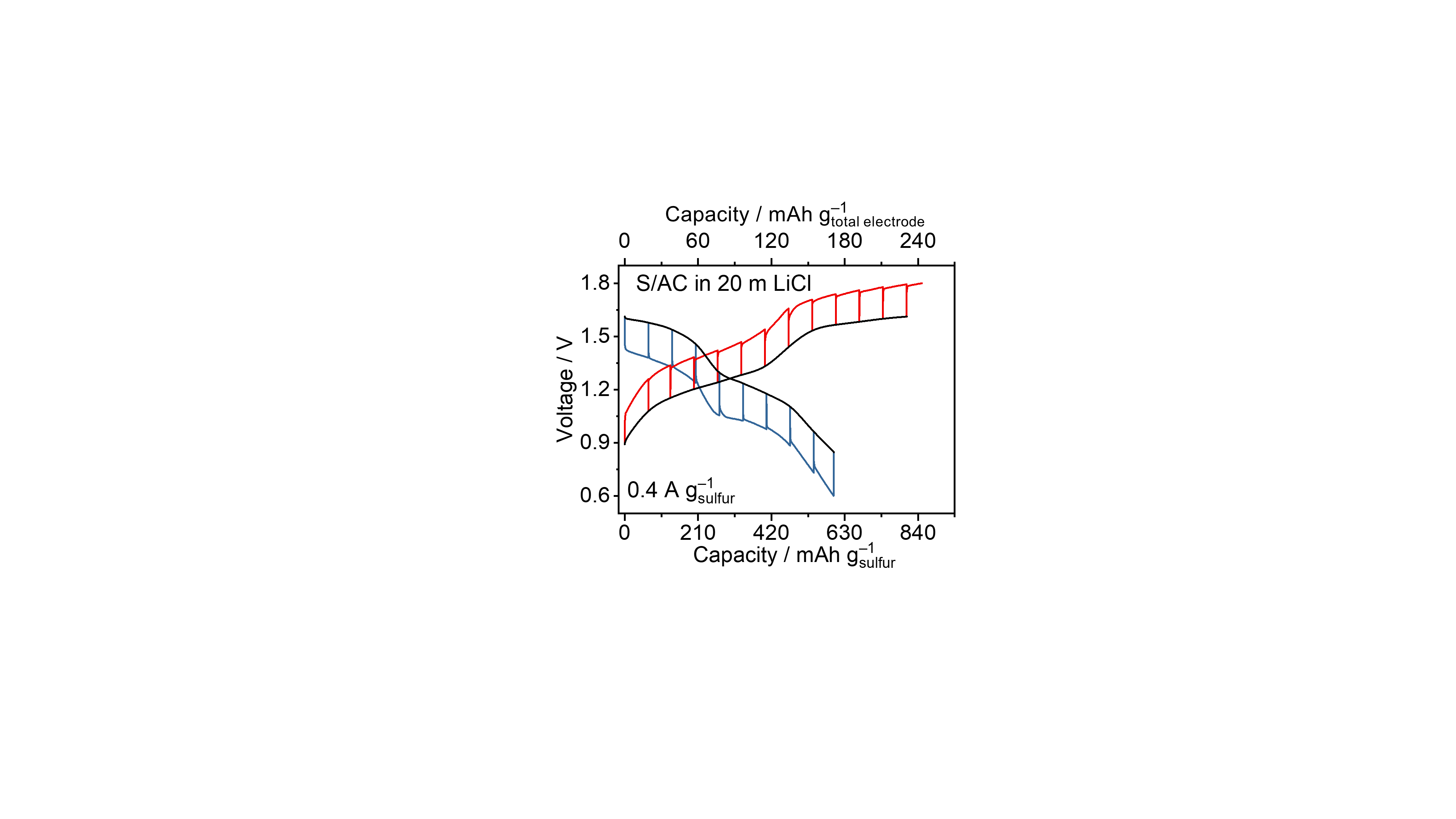


**Figure S26.** The GITT characterization of S/AC|20 m LiCl|I_2_/CC full cell at 0.4 A g^–1^. The voltage gaps are up to 170–200 mV during the most of charge/discharge process.

**Table S1**. Compositions of simulated electrolytes.

|  | 5 m LiCl | 20 m LiCl | WiBS |
| --- | --- | --- | --- |
| Number of H_2_O per box | 2200 | 2200 | 2200 |
| Number of LiNO_3_ per box | / | / | 280 |
| Number of LiCl per box | 200 | 800 | 600 |
| T / K | 298 | 298 | 298 |

**Table S2**. The comparison of various redox reactions at different current densities in S/AC/MXene|WiBS|I_2_/CC cell.

|  | Different discharge states | | | | | | | | | |
| --- | --- | --- | --- | --- | --- | --- | --- | --- | --- | --- |
|  | Cl^0^/Cl^–^ | | | | | I^+^/I^0^ | | | | |
| Current density / mA g^–1^ | 0.4 | 0.6 | 0.8 | 1.0 | 1.2 | 0.4 | 0.6 | 0.8 | 1.0 | 1.2 |
| Capacity contribution | 52% | 45% | 36% | 25% | 15% | 48% | 55% | 64% | 75% | 85% |
| Plateau voltage / V | 1.54 | 1.44 | 1.38 | 1.33 | 1.25 | 1.10 | 1.02 | 0.99 | 0.95 | 0.93 |

**Table S3**. Summary of fitted results from EIS spectra of different cycles in different ASDBs.

| Different ASDBs | Cycle | *R_s_* / Ω | *R_f_* / Ω | *R_ct_* / Ω |
| --- | --- | --- | --- | --- |
| S/AC\|20 m LiCl\|I_2_/CC | 2^nd^ | 10.4 | 24.2 | 129.9 |
|  | 150^th^ | 21.8 | 325.8 | 153.4 |
| S/AC\|WiBS\|I_2_/CC | 2^nd^ | 11.2 | 21.0 | 110.9 |
|  | 150^th^ | 19.6 | 220.7 | 147.1 |
| S/AC/MXene\|WiBS\|I_2_/CC | 2^nd^ | 9.6 | 39.8 | 106.3 |
|  | 150^th^ | 16.1 | 120.4 | 134.3 |

**Table S4**. The comparison of electrochemical performances reported to the previous aqueous batteries based on the aqueous chalcogen and halogen conversion chemistry.

| Battery systems | Voltage (V) | Mass ratio of cathode to anode | Capacity  (mAh g^–1^) | Specific Energy (Wh kg^–1^) | Ref. |
| --- | --- | --- | --- | --- | --- |
| PTCDA\|\|I_2_/CC | 1.05 | 1:2.5 | 106@0.5 A g^–1^ | 94.3 | ^[12]^ |
| S/C\|\|I_2_/CC | 1.24 | 2.5:1 | 128@0.25 A g^–1^ | 158.7 | ^[15]^ |
| Zn\|\|TPABr_3_ | 1.64 | / | 88.75@0.5 A g^–1^ | 142 | ^[16]^ |
| S/KB\|\|LiCoO_2_ | 1.64 | 4.51:1 | 119@0.2C | 195 | ^[13d]^ |
| S/KB\|\|LiMn_2_O_4_ | 1.60 | 6.9:1 | 84.5@0.2C | 135 | ^[13d]^ |
| Zn\|\|S/C | 0.50 | / | 136@0.1 A g^–1^ | 68 | ^[17]^ |
| Zn\|\|Cu_2_S | 1.15 | / | 221.7@0.1 A g^–1^ | 255 | ^[18]^ |
| S/C\|\|Ca_0.4_MnO_2_ | 1.29 | 1.6:1 | 86@0.2C | 110 | ^[13c]^ |
| S/C\|\|Mg_x_MnO_2_ | 1.20 | 2:1 | 53@0.2C | 66.4 | ^[13c]^ |
| S/C\|\|Al_x_MnO_2_ | 0.60 | 4:1 | 30@0.2C | 18 | ^[13c]^ |
| Ca\|\|Se/CMK–3 | 1.10 | / | 31@50 mA g^–1^ | 34 | ^[19]^ |
| S/MNC\|\|Na_0.44_MnO_2_ | 1.17 | / | 94.78@0.5C | 110.6 | ^[20]^ |
| S@CoS_2_–IL\|\|Na_0.44_MnO_2_ | 0.98 | 5.9:1 | 104.98@0.5C | 103 | ^[21]^ |
| Zn\|\|Te | 0.62 | / | 120@50 mA g^–1^ | 74.1 | ^[22]^ |
| S/AC/MXene\|\|I_2_/CC | 1.32 | 1:1 | 242@0.4 A g^–1^ | 304 | This work |

**Reference**

[1] M. Ghidiu, M. R. Lukatskaya, M.-Q. Zhao, Y. Gogotsi, M. W. Barsoum, *Nature* **2014**, 516, 78-81.

[2] A. M. Gaikwad, G. L. Whiting, D. A. Steingart, A. C. Arias, *Adv. Mater.* **2011**, 23, 3251-3255.

[3] Q. Zhao, Y. Lu, Z. Zhu, Z. Tao, J. Chen, *Nano Lett.* **2015**, 15, 5982-5987.

[4] K. Hongsirikarn, X. Mo, J. G. Goodwin, S. Creager, *J. Power Sources* **2011**, 196, 3060-3072.

[5] D. Ma, B. Hu, W. Wu, X. Liu, J. Zai, C. Shu, T. Tadesse Tsega, L. Chen, X. Qian, T. L. Liu, *Nat. Commun.* **2019**, 10, 3367.

[6] S. Plimpton, *Comput. Mater. Sci.* **1995**, 4, 361-364.

[7] L. Martínez, R. Andrade, E. G. Birgin, J. M. Martínez, *J. Comput. Chem.* **2009**, 30, 2157-2164.

[8] a) M. B. Singh, V. H. Dalvi, V. G. Gaikar, *RSC Adv.* **2015**, 5, 15328-15337; b) J. Zheng, G. Tan, P. Shan, T. Liu, J. Hu, Y. Feng, L. Yang, M. Zhang, Z. Chen, Y. Lin, J. Lu, J. C. Neuefeind, Y. Ren, K. Amine, L.-W. Wang, K. Xu, F. Pan, *Chem.* **2018**, 4, 2872-2882; c) Y. Zou, T. Liu, Q. Du, Y. Li, H. Yi, X. Zhou, Z. Li, L. Gao, L. Zhang, X. Liang, *Nat. Commun.* **2021**, 12, 170.

[9] T. Brezesinski, J. Wang, S. H. Tolbert, B. Dunn, *Nat. Mater.* **2010**, 9, 146-151.

[10] G. Liang, B. Liang, A. Chen, J. Zhu, Q. Li, Z. Huang, X. Li, Y. Wang, X. Wang, B. Xiong, X. Jin, S. Bai, J. Fan, C. Zhi, *Nat. Commun.* **2023**, 14, 1856.

[11] S. Lv, T. Fang, Z. Ding, Y. Wang, H. Jiang, C. Wei, D. Zhou, X. Tang, X. Liu, *ACS Nano* **2022**, 16, 20389-20399.

[12] Y. Zhang, S. Ying, Z. Ding, C. Wei, Q. Wang, C. Zhou, G. Zhou, X. Tang, X. Liu, *ACS Nano* **2023**, 17, 22656-22667.

[13] a) X. Liang, C. Hart, Q. Pang, A. Garsuch, T. Weiss, L. F. Nazar, *Nat. Commun.* **2015**, 6, 5682; b) Y.-S. Su, Y. Fu, T. Cochell, A. Manthiram, *Nat. Commun.* **2013**, 4, 2985; c) X. Tang, D. Zhou, B. Zhang, S. Wang, P. Li, H. Liu, X. Guo, P. Jaumaux, X. Gao, Y. Fu, C. Wang, C. Wang, G. Wang, *Nat. Commun.* **2021**, 12, 2857; d) C. Yang, L. Suo, O. Borodin, F. Wang, W. Sun, T. Gao, X. Fan, S. Hou, Z. Ma, K. Amine, K. Xu, C. Wang, *Proc. Natl. Acad. Sci. U.S.A.* **2017**, 114, 6197-6202.

[14] H. Sun, G.-L. Xu, Y.-F. Xu, S.-G. Sun, X. Zhang, Y. Qiu, S. Yang, *Nano Res.* **2012**, 5, 726-738.

[15] C. Wei, Y. Wang, Z. Ding, T. Fang, J. Song, Y. Zhang, S. Lv, X. Liu, X. Tang, *Adv. Funct. Mater.* **2023**, 33, 2212644.

[16] L. Gao, Z. Li, Y. Zou, S. Yin, P. Peng, Y. Shao, X. Liang, *iScience* **2020**, 23.

[17] W. Li, K. Wang, K. Jiang, *Adv. Sci.* **2020**, 7, 2000761.

[18] X. Wu, A. Markir, L. Ma, Y. Xu, H. Jiang, D. P. Leonard, W. Shin, T. Wu, J. Lu, X. Ji, *Angew. Chem. Int. Ed.* **2019**, 58, 12640-12645.

[19] R. Zhou, Z. Hou, Q. Liu, X. Du, J. Huang, B. Zhang, *Adv. Funct. Mater.* **2022**, 32, 2200929.

[20] M. Kumar, T. C. Nagaiah, *Energy Stor. Mater.* **2022**, 49, 390-400.

[21] M. Kumar, A. K. Padhan, D. Mandal, T. C. Nagaiah, *Energy Stor. Mater.* **2022**, 45, 1052-1061.

[22] Z. Chen, Q. Yang, F. Mo, N. Li, G. Liang, X. Li, Z. Huang, D. Wang, W. Huang, J. Fan, C. Zhi, *Adv. Mater.* **2020**, 32, 2001469.
